# Transcriptome of the dead: characterisation of immune genes and marker development from necropsy samples in a free-ranging marine mammal

**DOI:** 10.1186/1471-2164-14-52

**Published:** 2013-01-24

**Authors:** Joseph I Hoffman, Michael AS Thorne, Philip N Trathan, Jaume Forcada

**Affiliations:** 1 Department of Animal Behaviour, University of Bielefeld, Postfach 100131, Bielefeld, 33501, Germany; 2 British Antarctic Survey, Natural Environment Research Council, High Cross, Madingley Road, Cambridge, CB3 0ET, United Kingdom

**Keywords:** Transcriptome, Genomics, Non-model organism, *Post mortem*, Immune gene, Major Histocompatibility Complex (MHC), Microsatellite, Single Nucleotide Polymorphism (SNP), Marine mammal, Antarctic fur seal, *Arctocephalus gazella*, Pinniped

## Abstract

**Background:**

Transcriptomes are powerful resources, providing a window on the expressed portion of the genome that can be generated rapidly and at low cost for virtually any organism. However, because many genes have tissue-specific expression patterns, developing a complete transcriptome usually requires a 'discovery pool' of individuals to be sacrificed in order to harvest mRNA from as many different types of tissue as possible. This hinders transcriptome development in large, charismatic and endangered species, many of which stand the most to gain from such approaches. To circumvent this problem in a model pinniped species, we 454 sequenced cDNA from testis, heart, spleen, intestine, kidney and lung tissues obtained from nine adult male Antarctic fur seals (*Arctocephalus gazella*) that died of natural causes at Bird Island, South Georgia.

**Results:**

After applying stringent quality control criteria based on length and annotation, we obtained 12,397 contigs which, in combination with 454 data previously obtained from skin, gave a total of 23,096 unique contigs. Homology was found to 77.0% of dog (*Canis lupus familiaris*) transcripts, suggesting that the combined assembly represents a substantial proportion of this species' transcriptome. Moreover, only 0.5% of transcripts revealed sequence similarity to bacteria, implying minimal contamination, and the percentage of transcripts involved in cell death was low at 2.6%. Transcripts with immune-related annotations were almost five-fold enriched relative to skin and represented 13.2% of all spleen-specific contigs. By reference to the dog, we also identified transcripts revealing homology to five class I, ten class II and three class III genes of the Major Histocompatibility Complex and derived the putative genomic distribution of 17,121 contigs, 2,119 *in silico* mined microsatellites and 9,382 single nucleotide polymorphisms.

**Conclusions:**

Our findings suggest that transcriptome development based on samples collected *post mortem* may greatly facilitate genomic studies, not only of marine mammals but also more generally of species that are of conservation concern.

## Background

Massively parallel sequencing approaches such as Roche 454 and Illumina HiSeq are transforming the study of non-model organisms by dramatically increasing sequencing depth and coverage in return for a greatly reduced investment in time, labour and resources [1]. This has facilitated the development of transcriptomes, which provide access to the coding sequences of literally thousands of genes and can be mined for genetic markers for use in genome scans, quantitative trait loci mapping and various other applications [2,3].

mRNA sequencing is a particularly powerful approach for developing 'entry level' genomic resources for studying natural populations of non-model organisms, which are often compelling from ecological or evolutionary perspectives but poorly characterised genetically [4,5]. The resulting transcriptomes can in turn be mined for Single Nucleotide Polymorphisms (SNPs) which have already proven powerful for gene mapping [6] and are likely to become increasingly important in conservation genetics since they allow the characterisation of population structure and genetic diversity with unprecedented resolution [7]. However, to exhaustively sample a given species' transcriptome usually requires animals to be sacrificed so that transcripts can be harvested from as many different organs as possible [5], with the spleen being a particularly important source of immune genes [8]. Although this is generally considered acceptable for small, tractable and highly abundant organisms such as many insects [9], molluscs [10] and even fish [11], it is less appropriate for species that are large, highly charismatic or threatened. One potential solution, supported by recent expression studies of human cadavers [12] and slaughtered domestic pigs [13], is to sequence tissues obtained shortly after animals have died of natural causes.

Marine mammals provide an interesting case in point, being large and charismatic but extremely difficult to study given that they spend most, if not all, of their time at sea. Unfortunately, many marine mammal populations are also severely depleted due to a combination of historical exploitation [14] and contemporary threats including bycatch and other fisheries interactions [15] and climate change [16]. These factors may explain why only two marine mammal EST libraries, in both cases developed from either skin or blood, have been published to date [17,18]. Nevertheless, marine mammals are strong candidates for generating transcriptomes from tissues collected *post mortem*, since colonially breeding pinnipeds tend to suffer from high mortality rates and occasional mass mortality events [19,20], while many cetacean species are routinely stranded ashore *en masse*[21].

The Antarctic fur seal (*Arctocephalus gazella*) is a highly sexually dimorphic pinniped that breeds in crowded rookeries where adult males compete fiercely for access to females [22]. On Bird Island, South Georgia, a colony has been studied since the early 1980s with an aerial walkway providing unprecedented access for tissue sampling and the collection of detailed daily behavioural observations including metre-resolution locations of every adult male sighted ashore [23]. An ongoing molecular study spanning almost two decades has shown that most, if not all, pups are conceived on land [24] and that heterozygosity measured at nine microsatellites correlates with virtually every component of male reproductive success so far analysed including territory holding ability [25], body size [26] and attractiveness to females [27]. However, a paucity of genomic resources for this species, as well as for pinnipeds in general, constrains our ability to understand the underlying mechanisms [28].

To circumvent this problem, we recently developed a partial transcriptome assembly by 454 sequencing non-destructively obtained skin biopsy samples from twelve individuals of this species [29]. In a subsequent pilot study, we showed that it was possible to obtain polymorphic microsatellites targeted towards candidate genes related to immunity and growth by selecting loci that appear variable *in silico*[30]. We also exploited homology to the dog (*Canis lupus familiaris*) genome to map transcripts to specific chromosomes, allowing the development of a genome-wide distributed panel of 104 validated, polymorphic SNPs [31].

Although our initial transcriptome was more than adequate for many purposes, immune-related transcripts were not as numerous as originally hoped for, probably due to our having been restricted to the use of skin samples. Moreover, many of the immune-related contigs that we were able to construct had too little depth of sequence coverage to allow SNPs to be called with high confidence. This hampers the development of SNPs within candidate immune genes, a classic example of which, the Major Histocompatibility Complex (MHC), was recently found to be a remarkably strong predictor of survivorship to adulthood in the closely related grey seal, *Halichoerus grypus*[32].

Here, we 454 sequenced tissues obtained from six different organs (testis, heart, spleen, intestine, kidney and lung) obtained at necropsy from nine adult male fur seals that died of natural causes. We constructed tissue-specific assemblies to compare the representation of immune-related transcripts, including those showing homology to the MHC, and exploited comparative genomics with the dog to map the putative locations of transcripts and *in silico* derived genetic markers.

## Results

### RNA yields from necropsy samples

Yields of total RNA averaged 398 ng/μl per sample and varied significantly by tissue type (Table 1, Two way ANOVA, *F* = 6.33, d.f. = 1, *P* = 0.015), with the greatest yields coming from the testis and the lowest from the heart. The time elapsed between death and necropsy did not explain significant variation in RNA concentration (Two way ANOVA, *F* = 1.09, d.f. = 1, *P* = 0.302), although weak negative correlations were observed between RNA yield and the time since death for all but one of the tissue types.

**Table 1:** Tissue samples collected after death from nine adult male Antarctic fur seals

**Male ID**	**Approximate time between death and necropsy (hours)**	**Whole RNA concentration (ng/μl)**					
		Testis	Heart	Spleen	Intestine	Kidney	Lung
Agaz0820	1	900	160	1467	105	550	500
Agaz0821	13	1888	180	1100	650	667	160
Agaz1001	18	578	90	200	34	4	180
Agaz1006	7	432	160	200	115	175	381
Agaz1007	8	500	135	389	160	514	160
Agaz1008	8	1000	75	375	165	446	85
Agaz1009	20	938	115	160	200	408	338
Agaz1010	20	737	120	563	596	534	89
Agaz1011	30	–	64	737	333	175	115

A testis sample was unavailable from Agaz1011 due to the carcass having been scavenged by giant petrels (*Macronectes giganteus* and *M. halli*).

### Sequence data generated

**Figure 1:**
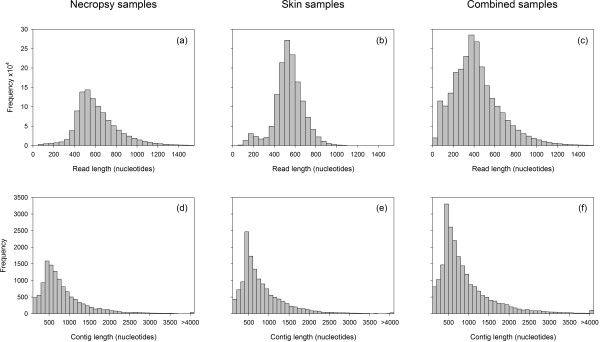
Read (a–c) and contig (d–f) length distributions for the necropsy (a and d), skin (b and e) and combined (c and f) libraries.

The cDNA library was subject to a full 454 sequencing run, yielding 1,046,221 reads of mean length 618bp and totalling 347,354,924bp (see Table 2 for details). Although we obtained only 72.5% of the total number of reads previously generated by a full 454 run based on skin biopsy samples, read lengths were significantly longer (Figure 1, unpaired *t*-test, *P* < 2.2e^-16^) due to our having switched from FLX to FLX+ sequencing chemistry.

**Table 2:** Summary of 454 sequence data obtained from the Antarctic fur seal

	**Testis**	**Heart**	**Spleen**	**Intestine**	**Kidney**	**Lung**	**Skin**
Number of reads	97,799	38,332	275,366	167,555	336,223	130,946	1,443,397
Mean read length	823	689	597	567	546	740	542
Total base pairs (bp)	33,342,585	12,996,565	90,644,969	57,138,133	108,713,181	44,519,491	412,933,200

Results are given separately for each type of tissue obtained at necropsy as well as for a normalised cDNA library previously generated from skin biopsy samples [29].

### Transcriptome assemblies

Assembly of the necropsy data consecutively with Newbler and CAP 3 [33] to minimise redundancy resulted in a total of 14,362 contigs. Selection based on a minimum contig length of 500bp or, in the case of smaller contigs, for annotation e-values below 1e^-10^ resulted in 12,397 contigs (86.3%) being retained for further analysis. Added to these reads was another set of sequencing on skin biopsy samples described previously [29]. These data were assembled using the same bioinformatic pipeline into 20,330 contigs, of which 14,271 (70.2%) were retained after filtering on size and annotation. Besides the assemblies of these two sets of sequences, allowing for direct comparison, a combined transcriptome using both sets of reads was constructed. This comprised 30,765 contigs, of which 23,096 (75.1%) were retained for the final transcriptome database. Contig lengths were qualitatively very similar among the three assemblies (Figure 1) and the combined assembly had an average contig length of 971bp.

**Figure 2:**
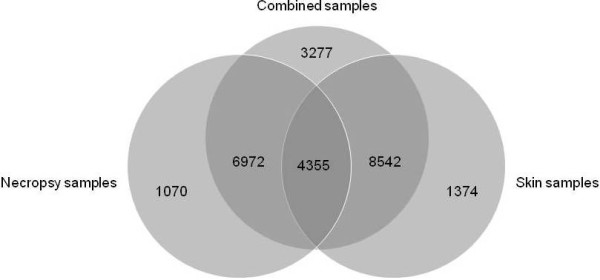
**Venn diagram showing the differential representation of transcripts among the necropsy, skin and combined assemblies (see Results for details).** The numbers of contigs represented within each portion of the diagram are shown. Light grey portions correspond to contigs observed within a single assembly, mid grey portions to contigs common to two different assemblies, and the dark grey portion corresponds to contigs shared among all three assemblies.

To explore the differential representation of transcripts among the three assemblies, we BLASTed the combined assembly against those derived from necropsy and skin. A highly stringent e-value threshold of 1e^-100^ was employed to ensure that any matches are indicative of near-perfect similarity. As expected, the majority of contigs from the combined assembly revealed strong homology to contigs from the necropsy (*n* = 11,327, 49.0%) and skin (*n* = 12,897, 55.8%) assemblies, with the total number of overlapping contigs from all three sets being 19,869 (86.0% of the combined set). However, only 4,355 of the combined assembly contigs (18.9%) matched both necropsy and skin, implying a limited degree of overlap between the latter two assemblies (Figure 2).

### Functional annotation

**Figure 3:**
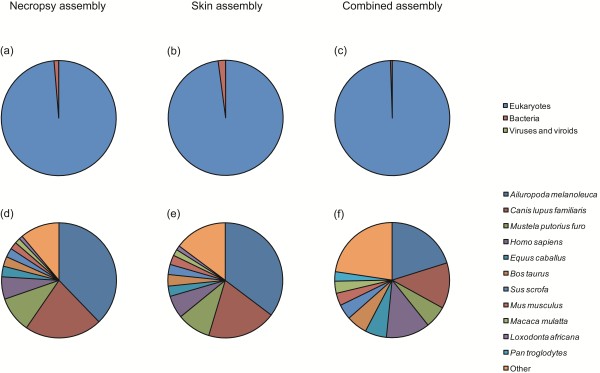
**Taxonomic distribution of top BLAST hits for the necropsy (a and d), skin (b and e) and combined (c and f) assemblies, classified into eukaryotes, bacteria or viruses (a**–**c) and according to the top ten species represented (d**–**f).**

Sequence similarity searches to the non-redundant (nr) database produced matches for a total of 15,073 contig sequences (65.3% of the total 23,096 contigs). The majority of sequences showed top matches to eukaryotic species, with the remaining matches involving mostly pathogenic bacteria (see Figure 3a–c). Reassuringly, the proportion of bacterial matches was actually lower in the necropsy assembly than in the skin assembly (0.5% versus 2.1%), suggesting that we managed to avoid contaminating the samples during the necropsy procedure. Figure 3d–f shows the taxonomic breakdown of the top matches obtained for the three assemblies by species. Although there was minor variation among the assemblies, the top ten matches were consistently to mammalian species, with the giant panda (*Ailuropoda melanoleuca*) and the dog being the two most frequently represented.

**Figure 4:**
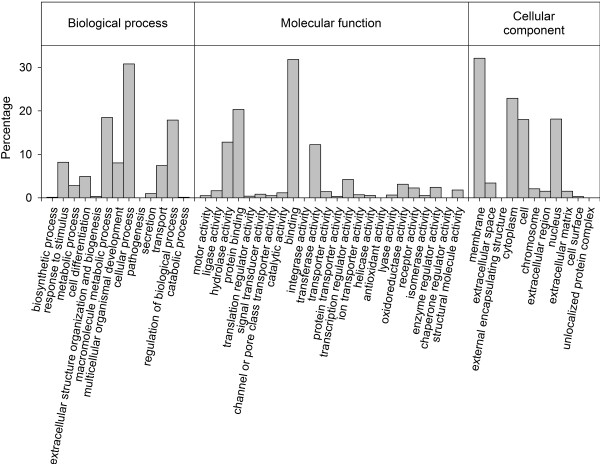
Gene ontology classifications for the combined assembly, summarized for three major categories: biological process, molecular function and cellular component.

Using standard GO annotation, contigs from the combined assembly were next classified according to three major functional categories: biological process, molecular function and cellular component. Molecular function made up the majority of GO designations (*n* = 53,189) followed by biological processes (*n* = 46,947) and then cellular component (*n* = 32,140). These data are summarised in Figure 4, which reveals a functionally diverse range of annotation terms. Summaries generated separately for the necropsy and skin assemblies were qualitatively very similar and are therefore not shown.

**Figure 5:**
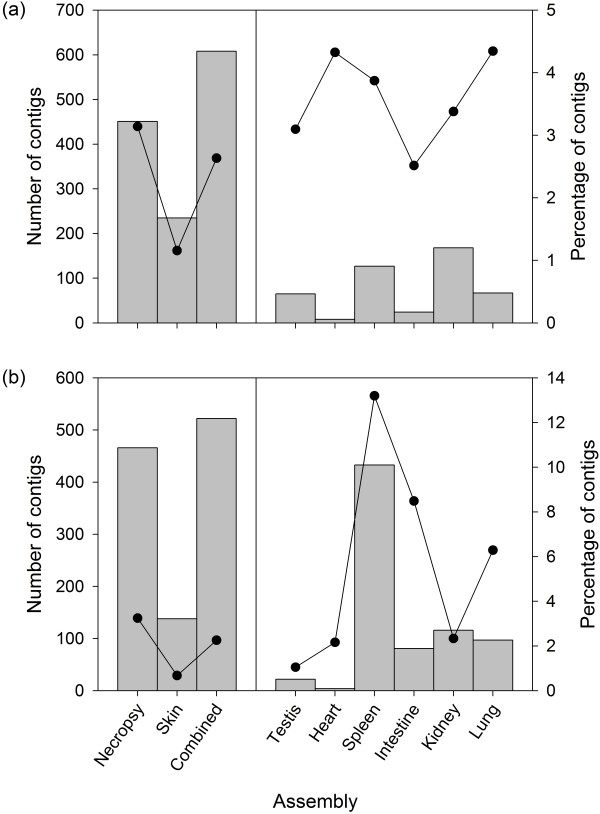
Numbers and percentages of contigs from each assembly, denoted by vertical bars and points respectively, with GO annotations relating to (a) apoptosis; and (b) immunity.

To discount the possibility that the necropsy assembly could be dominated by genes involved in cell death, we next interrogated the three assemblies for contigs with GO-codes relating to apoptosis. Figure 5a shows that, although contigs representing apoptosis-related genes are almost twice as numerous in the necropsy assembly than in the skin assembly, their overall percentages are low in all three assemblies. Furthermore, relatively little variation was found in the numbers and percentages of apoptosis-related transcripts among the different types of tissue obtained at necropsy.

We next exploited GO annotation terms to extract a total of 521 immune-related contigs, which are listed in Additional file 1: Table S1. These had a mean length of 854bp and contained a total of 1,471 SNPs (see subsequent section for SNP discovery). Associated GO terms were diverse, including 'immune system process', 'immune response', chemokine activity', 'antigen processing and presentation', and 'T cell selection'. As anticipated, immune-related contigs were over three times more numerous in the necropsy assembly than in the skin assembly, despite the overall amount of 454 sequence data being lower (Figure 5b). This is largely attributable to our having included spleen tissues in the necropsy assembly, which alone yielded 433 contigs with GO annotations relating to immunity, accounting for 13.2% of all spleen-specific contigs. Moreover, in contrast to the other tissues, four of the top ten most abundant transcripts identified in the spleen were immune related (Additional file 2: Table S2), implying relatively high levels of expression.

### Comparative genomics between the fur seal and dog

**Figure 6:**
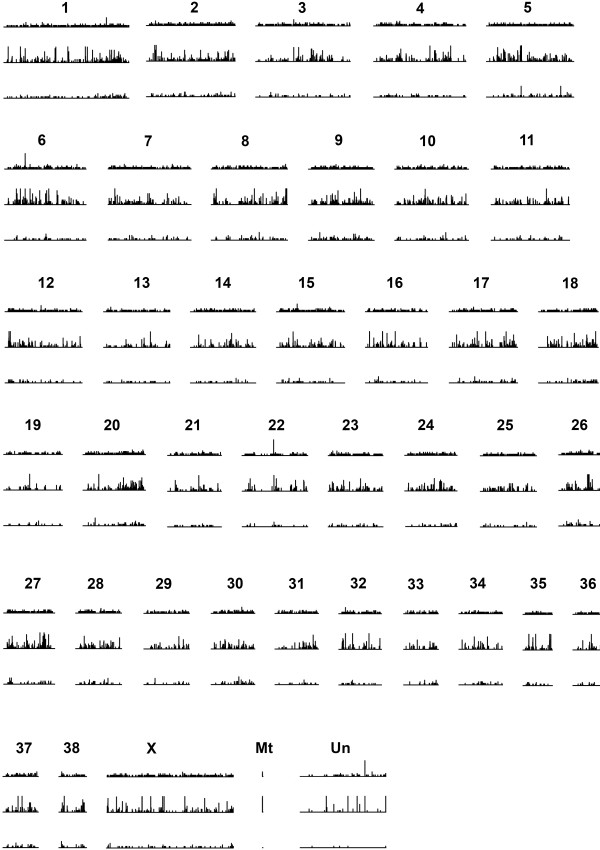
**Chromosomal distribution of the numbers of contigs (top row), SNPs identified using the Newbler mapping program (middle row) and microsatellites (bottom row) inferred by mapping to the dog (*Canis familiaris*) genome (see** Methods **for details).**

To shed light on the genomic distribution of the 454 sequence data, we mapped unassembled reads and contig sequences to the dog genome. A total of 1,521,212 individual reads (61.1%) successfully mapped, with 50.1% of these uniquely mapping to a single chromosome. A strong positive relationship was found between the number of reads mapping to a given chromosome and the length of that chromosome in the dog (r^2^ = 0.73, *n* = 39, *P* < 0.0001), indicating a fairly even genomic distribution. As anticipated, the number of contigs from the combined assembly mapping to the dog genome was highly dependent on the e-value threshold applied in the BLAST analysis. This varied from 22,424 (97.1%) with a threshold of 1e^-4^ through 17,121 (74.1%) with a threshold of 1e^-50^ to 4,118 (17.8%) with a threshold of 0. The proportion of contigs mapping to more than one chromosome also decreased with increasing stringency, (50.8%, 77.6% and 89.9% for 1e^-4^, 1e^-50^ and 0 respectively). To provide an optimal balance between the number of mapped contigs and the stringency required for uniqueness, we therefore chose an e-value of 1e^-50^ for all subsequent analyses when mapping between fur seal contigs and the dog genome. Using these data, we obtained a similar pattern to the reads, in which the number of mappings correlated positively with chromosome length in the dog (r^2^ = 0.78, *n* = 39, *P* < 0.0001). The detailed genomic distribution of contigs inferred by mapping to the dog is shown in Figure 6.

Another question that can be addressed through comparative genomics is the extent to which a given collection of transcripts represents an exhaustive transcriptome. To evaluate the completeness of the fur seal transcriptome, we therefore explored homology between the combined assembly and the complete set of dog transcripts using a standard e-value threshold of 1e^-10^. A total of 14,435 (62.5%) fur seal contigs showed homology to 19,691 of the 25,559 (77.0%) dog transcripts, suggesting that the combined assembly represents a substantial proportion of the fur seal transcriptome.

We also exploited homology between the fur seal and dog to explore the tissue-specific representation of transcripts revealing homology to 21 different canine MHC genes. The mapping data were consistent with the expression of five class I genes, ten class II genes and three class III genes (Table 3). The number of reads per million mapping to these genes varied significantly by MHC class (Two way ANOVA; *F* = 6.16, d.f. = 1, *P* = 0.014) but not by tissue (Two way ANOVA; *F* = 0.13, d.f. = 1, *P* = 0.723). Nevertheless, total number of reads per million was highest for class I and II genes in the spleen, whereas for class III genes this was highest in the lung. The skin also had by far the lowest representation of MHC-homologous transcripts, with only 663 reads per million in comparison to 8330 reads per million for spleen.

**Table 3:** Numbers of reads per million mapping to known canine MHC genes, summarised for the various tissues plus the combined assembly (see Methods for details)

**Class**	**Gene**	**Reference**	**Chromosome in dog**	**Tissue**							**Combined**
				**Heart**	**Intestine**	**Kidney**	**Lung**	**Skin**	**Spleen**	**Testis**	
I	DLA-64		ENSCAFG00000000500	12	78.4	257.1	324.8	390.2	85.2	687.6	61.5	240.7
I	DLA-79		ENSCAFG00000008234	18	0.0	0.0	14.9	0.0	15.6	3.6	0.0	10.5
I	DLA-88 Precursor		ENSCAFG00000000487	12	0.0	269.1	268.2	451.5	43.6	654.8	61.5	210.3
I	DLA-88		ENSCAFG00000000492	12	78.4	191.4	283.1	550.9	1.0	753.0	71.7	207.8
I	Tapasin		ENSCAFG00000000972	12	0.0	41.9	110.2	91.8	2.1	149.2	30.7	50.8
II	BTNL2		ENSCAFG00000000797	12	0.0	0.0	0.0	0.0	0.0	0.0	0.0	0.0
II	CD74		ENSCAFG00000018101	4	52.3	71.8	143.0	145.4	0.0	338.3	41.0	88.7
II	DLA-DRA		ENSCAFG00000000803	12	26.1	59.8	134.1	298.4	165.2	516.6	20.5	198.3
II	DQ Alpha precursor		ENSCAFG00000000812	12	0.0	6.0	65.6	168.3	83.1	247.4	10.2	96.7
II	HLA-DMA		ENSCAFG00000000848	12	0.0	0.0	3.0	0.0	1.0	7.3	0.0	2.0
II	HLA-DOA		ENSCAFG00000000896	12	0.0	0.0	0.0	0.0	0.0	3.6	20.5	1.5
II	HLA-DOB		ENSCAFG00000000819	12	0.0	0.0	0.0	0.0	0.0	0.0	0.0	0.0
II	HLA-DPB1		ENSCAFG00000000900	12	0.0	0.0	3.0	0.0	3.1	0.0	0.0	2.0
II	HLA-DQB1		ENSCAFG00000000814	12	0.0	17.9	26.8	53.6	6.2	54.6	10.2	20.4
II	HLA-DRB1		ENSCAFG00000000806	12	52.3	59.8	83.4	336.7	40.5	232.8	51.2	95.7
II	IK cytokine		ENSCAFG00000005869	2	52.3	6.0	62.6	38.3	128.9	36.4	61.5	84.2
II	RFX3		ENSCAFG00000002053	1	0.0	47.8	8.9	0.0	0.0	0.0	61.5	8.5
III	BAG6		ENSCAFG00000000575	12	130.7	131.6	122.2	122.4	4.2	80.0	706.9	89.2
III	HSP70		ENSCAFG00000000641	12	2117.7	998.7	2413.4	7789.6	83.1	4565.6	624.9	1730.3
III	LTA		ENSCAFG00000000515	12	0.0	0.0	0.0	0.0	0.0	0.0	0.0	0.0
III	TNF		ENSCAFG00000000517	12	0.0	0.0	0.0	7.7	0.0	0.0	0.0	0.5
				Total class I	156.9	759.5	1001.1	1484.5	147.6	2248.2	225.4	720.1
				Total class II	183.0	269.1	530.4	1040.7	428.1	1437.0	276.6	598.0
				Total class III	2248.4	1130.2	2535.6	7919.7	87.3	4645.6	1331.9	1820.0
				Grand total	2588.3	2158.8	4067.1	10444.8	663.0	8330.8	1833.9	3138.1

### Molecular marker discovery

**Figure 7:**
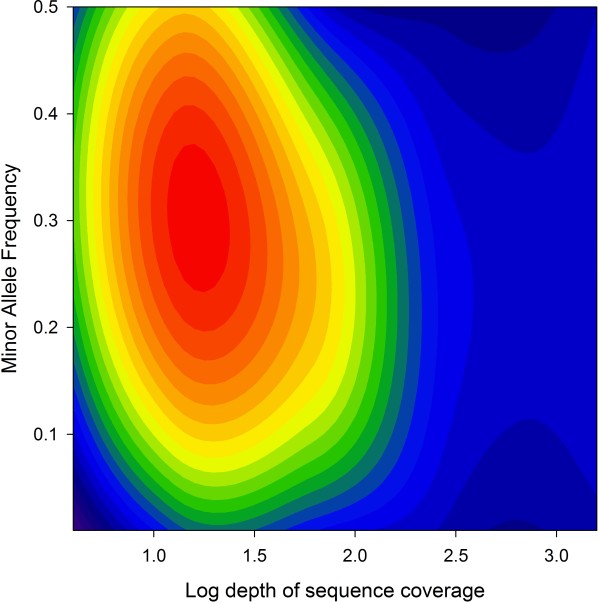
**Variation in the number of SNPs discovered in the combined assembly with Minor Allele Frequency (MAF) and depth of sequence coverage.** Local regression was used to fit a smoothed spline to the raw dataset, comprising 14,574 SNPs identified using the Newbler mapping program, implemented using the ‘locfit’ package in R. The area shaded in red indicates the parameter space associated with the greatest numbers of SNPs discovered.

Interrogating the combined assembly for perfect microsatellites with at least five repeat units identified 2,592 loci, 1,895 (73.1%) of which comprised dinucleotides, 561 (21.6%) trinucleotides and 92 (3.5%) tetranucleotides. These were located within 2131 different contigs, of which 1,185 (55.6%) were functionally annotated with respect to the nr database and 1,765 (82.8%) revealed significant matches to locations within the dog genome. To allow direct comparison with results previously obtained from the skin transcriptome assembly [29], we carried out an initial round of SNP discovery using the program SWAP454. Applying a 'strict' criterion in which SNPs were accepted when at least 33% of the reads differed from the reference sequence and the minor allele was observed at least eight times, a total of 1,585 SNPs were identified. Applying a 'relaxed' criterion that required only 10% of the reads to differ from the reference sequence and two copies of the minor allele to be present, the number of SNPs increased to 11,454. Next, to circumvent dependency on mapping the raw sequence reads to a reference assembly, we used the Newbler mapping program, identifying a total of 14,574 SNPs with high confidence. These were located within 5,205 different contigs, of which 3,617 (69.5%) were functionally annotated with respect to the nr database and 4,028 (77.4%) revealed significant matches to locations within the dog genome. To explore the corresponding SNP parameter space, we fitted a local regression to the raw data. Figure 7 reveals a clear peak in the number of SNPs identified at a MAF of around 0.3 and a log depth of coverage of approximately 1.2, which corresponds to a respectable 16x depth of coverage. The inferred genome-wide distributions of microsatellites and SNPs identified by the Newbler mapping program are shown in Figure 6.

## Discussion

Transcriptome sequencing has become an increasingly popular means of developing genomic resources for non-model organisms. However, to sample a transcriptome exhaustively usually requires animals to be sacrificed so that mRNA can be harvested from multiple tissues. To circumvent this problem in a natural population of Antarctic fur seals, we 454 sequenced cDNA from various tissues obtained at necropsy from nine adult males that died of natural causes. This appears to have been remarkably successful, leading to measurable increases in the number and diversity of transcripts characterised, a greater representation of transcripts involved in immunity, and many more genetic markers discovered.

It is important to acknowledge that this study is not the first to apply high throughput sequencing to tissues obtained after death. For example, Cherel et al. [13] recently explored links between gene expression and meat quality traits in recently slaughtered pigs, and Kang et al. [12] characterised the developmental progression of gene expression patterns in the human brain. However, such studies are few and far between, and tend to focus on humans and their companion species. Our study is novel in that we have harvested multiple organs from animals dying of natural causes in a natural population of a non-model organism in order to generate an exhaustive as possible *de novo* transcriptome assembly.

Given that some of our samples were obtained from adult males that had died up to thirty hours prior to necropsy (Table 1), we initially held concerns over the quantity of the RNA that could be extracted and whether this could have downstream impacts on the amount of resulting 454 sequence data. As anticipated, significant variation was found in the amount of total RNA that could be extracted from the different tissues, the more fibrous heart yielding the lowest average concentration despite our having used a bead mill. However, there was no clear relationship between RNA yield and the time elapsed between death and necropsy. Moreover, 454 read lengths were actually longer on average than previously obtained for the skin transcriptome. Although this is almost certainly due to our having switched from FLX to FLX+ sequencing chemistry, it nevertheless appears that using RNA from recently dead animals did not have a major detrimental impact on sequence length.

An important caveat to the above is that organs were harvested shortly after death (1–30 hours, mean = 13.9 hours) in order to minimise the risk of RNA degradation. Moreover, the prevailing climate at Bird Island during the austral summer is cool (usually between 1.5 and 4.5 degrees centigrade). It remains unclear how much high-quality RNA will be retained in carcasses at more advanced stages of decomposition, nor how dependent RNA degradation will be upon ambient temperature, making it difficult to extrapolate to other study systems. To evaluate this in fur seals would require significant further effort, since carcasses would need to be repeatedly sampled at different time intervals, generating multiple cDNA pools for sequencing. Moreover, such an experiment would ideally evaluate the importance of factors such as carcass size and prevailing environmental conditions.

For the previous assembly based on skin samples [29], we employed the cDNA option within Roche Newbler assembler version 2.3. However, a recent paper comparing the merits of five different assemblers found that this program performed poorly, generating the assembly with the greatest sequence redundancy and lowest proportion of mappings to reference sequences [34]. In contrast, a pre-release version of Newbler 2.5 was identified as the joint top performing assembler, with further improvements being obtained through the concurrent use of more than one assembler. To obtain the best possible quality transcriptome, we therefore assembled our data consecutively with Newbler 2.5 and CAP 3. After filtering on the basis of size and annotation, we obtained 23,096 contigs, which is considerably more than the equivalent value of 14,271 obtained when the original skin data were re-assembled and filtered based on the same criteria. Taken together with the limited degree of overlap between the necropsy and skin assemblies, this suggests that our improved transcriptome is larger and contains numerous transcripts that had not previously been described. This is consistent with previous studies that have found tissue-specific patterns of gene expression [35,36].

Quality filtering for size and annotation led to many small contigs being discarded from the final assembly. However, this was reflected in a greatly improved rate of annotation (65.3% of contigs yielded BLAST hits with an e-value cutoff of 1e^-10^) relative to the original skin transcriptome (47.0% of contigs yielded BLAST hits with an e-value cutoff of 1e^-4^). As found previously, the majority of matches were to vertebrates, with the giant panda and the dog being the two most frequently represented species. Interestingly, more matches were obtained to the panda than the dog for the necropsy and skin assemblies, but this pattern was reversed for the combined assembly. This difference may reflect the fact that the combined assembly contains substantially more larger transcripts than either the necropsy or skin assemblies (number of transcripts >1,000bp in length = 7,780, 3,789 and 4,320 respectively) due to the greater total number of reads available.

Reassuringly, the proportion of BLAST matches to bacterial sequences was actually lower for the necropsy than the skin assembly (0.5% versus 2.1%). This not only implies that we managed to avoid contaminating the samples during the necropsy procedure, but may also indicate that the seals we necropsied were not suffering from heavy bacterial infections at the time of death. The difference between the two assemblies if genuine would also be consistent with a previous study that isolated a diverse bacterial assemblage from fight wounds and lesions in adult males of this species [37]. Thus, the greater representation of bacterial sequences in skin could quite possibly reflect the relatively unsanitary conditions of life in a crowded fur seal breeding colony.

Although it was not critical that our transcriptome be perfectly representative of normal patterns of gene expression, we initially held concerns that a transcriptome derived from necropsy samples could be dominated by transcripts involved in cell death. To evaluate this possibility, we therefore interrogated each of the assemblies, including the tissue-specific assemblies, for GO terms relating to apoptosis. Contigs with apoptosis-related GO terms were around twice as numerous in the necropsy than in the skin assembly. However, their overall percentage was low, at only 2.6% for the combined assembly and rising to a maximum of 4.3% for the heart and lung tissues. Thus, although our data are consistent with a certain degree of up-regulation of genes involved in apoptosis, there is no suggestion of a major bias being present.

This study was partly motivated by the limited representation of immune-related transcripts in our original skin assembly, together with the fact that many of these had insufficient depth of sequence coverage to allow SNPs to be identified with high confidence. Using the same approach as above, but this time filtering for transcripts based on GO annotations relating to immunity, we identified a total of 521 immune-related contigs, almost four times more than in the skin assembly. This improvement appears largely attributable to the inclusion of samples from the spleen, which carried by far the greatest proportion of immune-related transcripts, at 13.2%. However, the main causes of death in adult male Antarctic fur seals are fight wounds and pneumonia, raising the possibility that at least some of these individuals could have been mounting an immune response to bacterial infection prior to death. This is difficult to judge given the low proportion of sequence matches to bacteria, but could potentially have contributed towards the elevated representation of immune-related transcripts in the necropsy assembly.

Through comparative genomics with the dog, we also analysed the tissue-specific representation of transcripts revealing homology to 21 different canine MHC genes. All but three of these genes, including class I, II and III genes, were represented in at least one tissue. Consistent with immune-related contigs being four times more common in the spleen, the total number of transcripts per million mapping to MHC class I and II genes was also highest for the spleen, followed by the lung. A contrasting pattern was obtained for the MHC class III, with the lung having almost twice as many transcripts per million mapping as the spleen. This may reflect the role of class III genes in mounting the immune response via innate immunity, inflammation and immunomodulation [38], processes that could conceivably be of greater importance in sensitive mucosal tissues such as the lung. Overall, we also found that MHC-related transcipts had the lowest representation in the skin, providing further justification for our having expanded and improved upon our original transcriptome.

Despite seals and dogs having diverged approximately 43 million years ago [39], the two genomes appear to have retained large syntenic blocks [40]. Sequence homology is also strong enough to allow the flanking sequences of most pinniped microsatellites to be mapped to unique locations in the dog [28,40], making the canine genome a powerful resource for comparative purposes. We therefore took previous seal studies a step further by inferring the genomic distribution of 1,521,212 reads (61.1%), 17,121 contigs (74.1%), 2,119 microsatellite loci (81.8%) and 9,382 SNPs (58.0%) by reference to the dog. Much as expected, the resulting distributions appear relatively even (Figure 6), as supported by a strong positive correlation between the number of contigs mapping to a given chromosome and the length of that chromosome in the dog. Moreover, although the mapping locations should be regarded as putative, we have reasons to believe that a substantial proportion will be correct. For example, Hoffman et al. [28] previously found that four microsatellite loci, described as putatively X-linked because they were homozygous in 84 males but carried the expected proportions of heterozygote genotypes in females, all mapped to the X chromosome in the dog [28]. Similarly, a SNP recently developed within a transcript revealing homology to mitochondrial NADH dehydrogenase revealed a pattern in which all individuals were called as homozygotes but for different alleles [31]. Nevertheless, to shed further light on synteny between the two genomes, it would be desirable to develop a high-density comparative linkage map. This is made feasible for the first time by the large number of genetic markers we have identified in this study (see below).

Mining the combined transcriptome assembly for microsatellites yielded marginally more markers than found in the original skin assembly (2,592 loci versus 2,271 loci respectively), although these numbers are not strictly comparable because different programs were used. A more obvious improvement was observed for SNPs, with SWAP454 identifying 2.5 times as many SNPs at the 'strict' level (1,585 versus 642) and 1.8 times more SNPs at the 'relaxed' level (11,454 versus 6,261). This is perhaps to be expected given the increased number and diversity of contigs and the improved depth of coverage, to which SNP discovery is particularly sensitive. Even more SNPs were identified by the Newbler mapping program (*n* = 14,574), with a clear peak in the parameter space corresponding to a MAF of around 0.3 and a depth of coverage of approximately 16x. This suggests that many of these SNPs may comfortably exceed the minimum selection criteria of at least three non-duplicate reads showing the variant and seven or more high-quality reads in total. One possible reason for the difference in the number of SNPs called by the two programs could be that SWAP454 relies on mapping the raw sequence reads back to a reference sequence, in this case the transcriptome assembly. This can lead to some loss of data due to incomplete mapping. Moreover, because the program only calls SNPs on the basis of reads that map reliably to a single contig, redundancy within the assembly, whether unintentional or due to the use of assembly methods that classify related contigs into 'isogroups' as constructed using the Newbler assembler, could potentially reduce the total number of SNPs called.

## Conclusions

By 454 sequencing samples obtained at necropsy, we have developed a greatly improved transcriptome assembly, thereby facilitating future evolutionary genetic studies of an important pinniped species, the Antarctic fur seal. We have also demonstrated that *post mortem* sampling provides a viable alternative to sacrificing animals, with positive implications for developing transcriptomes for charismatic and / or threatened species.

## Methods

### Animal sampling

Tissue samples were collected from nine freshly dead adult male Antarctic fur seals at Freshwater Inlet on Bird Island, South Georgia (54° 00Â´ S, 38° 02Â´ W) during the austral summer of 2010 / 2011. All specimens were known to have died within the last 30 hours by direct observation, and were transported immediately to the station laboratory to prevent scavenging by seabirds. The necropsies were conducted systematically within the shortest possible time window after death (see Table 1 for details). Tissue samples from all six organs were available for all but one of the animals, Agaz1011, due to this individual's testes having been scavenged by giant petrels (*Macronectes giganteus* and *M. halli*). Sampling equipment was sterilised using 95% ethanol between uses. Samples were transferred to RNAlater® and stored individually at −20°C for up to one month before being placed in a −80°C freezer for transport back to the UK.

### RNA isolation and cDNA generation

Approximately 10mg of each tissue sample was disrupted and homogenised by bead milling within a TissueLyserII (Qiagen). Total RNA was then extracted using a Qiagen RNeasy® mini kit following the manufacturer’s recommended protocols, with an optional on-column DNAse digestion step included. The resulting RNA pellets were resuspended in 50μl of RNAse-free water (Ambion) and quantified using PicoGreen (Invitrogen) fluorometry. RNA quantity and quality were also assessed visually by running a fraction of each isolate on a 1% agarose gel. Total RNA samples were pooled in equimolar ratios, as far as possible, for each of the tissue types. PolyA+ RNA was purified from total RNA using selection on Oligo-dT containing paramagnetic beads from the MicroPoly(A)PuristTM mRNA Purification Kit (Ambion, Life Technologies) according to the manufacturer’s instructions. 200ng mRNA of each tissue was used for cDNA Rapid Library construction according to 454/Roche FLX+ protocols. The individual tissue libraries were MID tagged, pooled and sequenced on a Roche Genome Sequencer FLX+ instrument using the GS FLX Titanium Sequencing Kit XL+ (Roche).

### Skin transcriptome data

Sequence data were also available from a previously published skin transcriptome [29]. This was based on a normalised cDNA library constructed from skin biopsy samples of twelve individuals and subjected to a full run on a Roche Genome Sequencer FLX Titanium, which yielded 1,443,397 454 reads of mean length 286bp.

### Sequence assembly

The pooled necropsy data and the previously sequenced skin biopsy data were separately assembled using Newbler (version 2.5.3), with a large genome style assembly followed by CAP 3 using default parameters [33] to reduce redundancy. A combined assembly using all the 454 data from both sets was then constructed in the same way to generate the best possible reference transcriptome for further analysis. After annotation using the Genbank non-redundant (nr) [41], swissprot [42] and dog transcript (ftp://ftp.ensembl.org/pub/release-67/fasta/canis_familiaris/ref) databases, we discarded all contigs less than 500bp in length that failed to reveal homology at an e-value less than 1e^-10^ in at least one of these databases.

### BLAST mapping and sequence annotation

To determine homology to known genes, Basic Local Alignment Search Tool (BLAST) searches [43] with a standard e-value cutoff of 1e^-10^ were used to query contig sequences against the GenBank nr and Swissprot databases. Gene Ontology (GO) mappings were determined using an in-house database taking the top five Swissprot matches. Immune and apoptosis related GO terms were then used to select fur seal transcripts with such matches.

### Comparative genomics with the dog

Using the combined assembly, four approaches were employed to explore sequence homology between the fur seal and dog. First, individual 454 reads were mapped to the dog genome (build 2.0) using Roche gsMapper version 2.3. The dog genome sequences in fasta format were obtained from ftp://ftp.ncbi.nih.gov/genomes/Canis_familiaris and comprised chromosomes 1 to 38 and X. Secondly, BLAST searches were carried out with the fur seal transcripts against each of the dog genome chromosomes. The highest score for a given transcript determined its placement along a chromosome. This provided the basis for positioning microsatellite and SNP loci (see below for marker discovery). Thirdly, BLAST searches were conducted comparing the assembled fur seal contigs against the set of canine nucleotide and peptide gene sequences, both annotated and abinitio, with an e-value cutoff of 1e^-10^.

We next compared *Canis familiaris* MHC genes for representation to the fur seal transcripts. These comprised 5, 12 and 4 class I, II, and III genes respectively (see Table 3 for details), identified from the literature [44,45] and through searches of the dog genome at Ensembl (http://www.ensembl.com). Newbler was used to map the 454 reads from the separate tissues against the selected MHC genes, including the associated variant transcripts. Following Ekblom et al. [35], we then used the number of transcripts mapping per million as a proxy for gene expression.

### Mining for molecular markers

The combined assembly was interrogated for microsatellite motifs using the program Phobos [46] to identify sequences containing perfect di-, tri- and tetranucleotide repeats with a minimum length of five repeat units. SNP detection was carried out using two programs. First, to provide a direct comparison with the numbers of SNPs previously obtained from the skin transcriptome [29], we applied the Swap454 pipeline [47]. This program first maps the raw reads back to the assembled contigs and then determines, while taking into account an error model for the 454 data, which positions are called as SNPs according to two user-specified thresholds. The first of these, 'MIN_RATIO' corresponds to the percentage of reads that differ from the reference sequence at a given position and the second, 'MIN_READS' to the number of copies present of the minor allele. We applied a 'strict' criterion to minimize the possibility of false positives arising from sequencing error and a 'relaxed' criterion to maximize the discovery of relatively infrequent alleles. For the former, MIN_RATIO was set to 0.33 and MIN_READS to 8, and for the latter MIN_RATIO was set to 0.1 and MIN_READS to 2. For comparison, we also used the Newbler mapping program (454 Life Sciences; http://www.454.com) to call SNPs that were deemed high confidence. For a SNP to be called in this way, there must be at least three non-duplicate reads showing the variant, with these reads being represented in both the forward and reverse directions, and at least seven reads with Phred quality scores of at least 20.

### Availability of supporting data

DNA sequences are available at the Genbank Sequence Read Archive (number: SRA064103).

## Competing interests

The authors declare that they have no competing interests.

## Authors’ contributions

JIH conceived and developed the project, prepared the RNA extracts, organised the sequencing, participated in data analysis and interpretation, and wrote the manuscript. MAST conducted the bioinformatics, participated in data analysis and interpretation, and contributed to manuscript preparation. JF conducted the field monitoring and necropsies of specimens and contributed tissue samples, and JIH, JF and PNT contributed funding towards the RNA extraction, cDNA synthesis and 454 sequencing. All authors commented upon and approved the final manuscript.

## Supplementary Material

Additional file 1: Table S1 List of immune-related transcripts identified in the combined assembly with corresponding BLAST hits against the nr database. Click here for file

Additional file 2: Table S2 The ten most commonly expressed sequences (in order of abundance) with associated BLAST matches. Data are presented sequentially for each type of tissue. Contig names correspond to the tissue-specific assemblies. Immune-related transcripts are denoted by an 'X'. Click here for file
