# Supplementary material for: Transcriptome of the dead: characterisation of immune genes and marker development from necropsy samples in a free-ranging marine mammal
Source: BMC Genomics. 2013 Jan 24;14:52. doi: 10.1186/1471-2164-14-52 (PMC3563519; doi:10.1186/1471-2164-14-52)
Supplement: Additional file 1: Table S1 — List of immune-related transcripts identified in the combined assembly with corresponding BLAST hits against the nr database. [file 1471-2164-14-52-S1.docx]

**Table S1:** List of immune-related transcripts identified in the combined assembly with corresponding BLAST hits against the nr database.

| Contig ID | Length (bp) | Number of SNPs | Gene description | Accession number | E-Value | Similarity | Score | Alignment length |
| --- | --- | --- | --- | --- | --- | --- | --- | --- |
| AgU000038_v1.1 | 5044 | 2 | interleukin-6 receptor subunit beta | XP_535239 | 1.78E-099 | 93 | 352.1 | 234 |
| AgU000077_v1.1 | 4343 | 9 | interferon-induced gtp-binding protein mx2 | XP_002925924 | 0 | 92 | 1261.9 | 702 |
| AgU000089_v1.1 | 4262 | 6 | complement c3 precursor | XP_002927812 | 0 | 95 | 2608.6 | 1411 |
| AgU000148_v1.1 | 3802 | 4 | complement component c7 precursor | XP_002922660 | 0 | 95 | 1432.2 | 842 |
| AgU000266_v1.1 | 3351 | 1 | complement component 4a (rodgers blood group) | XP_002929679 | 0 | 96 | 2014.6 | 1088 |
| AgU000270_v1.1 | 3343 | 3 | v-type proton atpase 116 kda subunit a isoform 4 | XP_539895 | 3.36E-102 | 97 | 352.4 | 173 |
| AgU000307_v1.1 | 3257 | 7 | serpin b12 | EFB24549 | 0 | 96 | 828.6 | 425 |
| AgU000335_v1.1 | 3200 | 9 | complement c1s subcomponent precursor | XP_002922266 | 0 | 96 | 1354.0 | 695 |
| AgU000384_v1.1 | 3098 | 2 | cd164 sialomucin | AER95801 | 4.31E-054 | 85 | 197.2 | 202 |
| AgU000398_v1.1 | 3070 | 3 | interleukin 1 type i | XP_002913539 | 1.42E-035 | 97 | 152.5 | 76 |
| AgU000502_v1.1 | 2898 | 11 | e3 ubiquitin-protein ligase trim22 | XP_002926117 | 0 | 92 | 874.4 | 470 |
| AgU000606_v1.1 | 2768 | 4 | tumor necrosis factor ligand superfamily member 10 | XP_002921635 | 0 | 97 | 542.7 | 286 |
| AgU000690_v1.1 | 2671 | 3 | transporter associated with antigen presentation partial | AFD62715 | 0 | 91 | 997.7 | 710 |
| AgU000704_v1.1 | 2657 | 1 | v-type proton atpase 116 kda subunit a isoform 3 | XP_540812 | 0 | 94 | 1339.7 | 829 |
| AgU000777_v1.1 | 2577 | 0 | atlastin gtpase 3 | CAB56010 | 0 | 96 | 594.0 | 331 |
| AgU000904_v1.1 | 2459 | 0 | complement factor b | XP_002929675 | 0 | 96 | 1493.8 | 768 |
| AgU000960_v1.1 | 2417 | 1 | cd97 antigen precursor | XP_002921043 | 0 | 86 | 992.6 | 624 |
| AgU001054_v1.1 | 2350 | 1 | stromal cell-derived factor 1 precursor | XP_003903642 | 8.30E-044 | 97 | 162.5 | 93 |
| AgU001099_v1.1 | 2317 | 1 | lactotransferrin isoform 1 | XP_541903 | 0 | 92 | 1245.0 | 689 |
| AgU001123_v1.1 | 2294 | 8 | leukocyte immunoglobulin-like subfamily a (with tm domain) member 1 | ACN73222 | 2.08E-138 | 88 | 318.9 | 193 |
| AgU001155_v1.1 | 2276 | 4 | deleted in malignant brain tumors 1 | XP_544052 | 0 | 90 | 854.0 | 498 |
| AgU001171_v1.1 | 2267 | 0 | interferon regulatory factor 2 | XP_001491848 | 0 | 98 | 652.1 | 337 |
| AgU001221_v1.1 | 2244 | 1 | interferon-induced protein with tetratricopeptide repeats 5 | EFB14122 | 5.78E-138 | 98 | 426.8 | 221 |
| AgU001310_v1.1 | 2193 | 1 | fc fragment of low affinity receptor | EFB26441 | 7.45E-126 | 88 | 385.2 | 228 |
| AgU001466_v1.1 | 2115 | 4 | bactericidal permeability-increasing 2 | XP_002921578 | 0 | 96 | 969.5 | 507 |
| AgU001475_v1.1 | 2102 | 1 | integrin alpha-d | XP_848776 | 0 | 93 | 804.3 | 470 |
| AgU001556_v1.1 | 2083 | 0 | complement component 4 binding alpha | XP_002928725 | 0 | 90 | 1035.8 | 604 |
| AgU001569_v1.1 | 2080 | 0 | inhibitor of nuclear factor kappa-b kinase subunit alpha | XP_002924321 | 0 | 97 | 618.2 | 309 |
| AgU001690_v1.1 | 2030 | 4 | sequestosome 1 | XP_003900678 | 0 | 91 | 678.7 | 438 |
| AgU001704_v1.1 | 2020 | 2 | myeloid cell nuclear differentiation antigen | XP_002927317 | 0 | 78 | 654.4 | 475 |
| AgU001734_v1.1 | 2013 | 0 | tumor necrosis factor receptor superfamily member 3 precursor | XP_002922295 | 0 | 94 | 721.1 | 428 |
| AgU001787_v1.1 | 1994 | 2 | interferon regulatory factor 1 | XP_002912943 | 0 | 99 | 589.3 | 321 |
| AgU001789_v1.1 | 1991 | 0 | t-cell surface glycoprotein cd4 precursor | ABS50090 | 1.10E-081 | 90 | 276.6 | 193 |
| AgU001802_v1.1 | 1994 | 0 | phosphoinositide-3- regulatory subunit 1 | BAE37914 | 5.03E-020 | 100 | 95.5 | 45 |
| AgU001849_v1.1 | 1969 | 0 | dna cross-link repair 1c (pso2 cerevisiae) | XP_002929927 | 2.38E-160 | 87 | 488.8 | 369 |
| AgU001879_v1.1 | 1957 | 0 | complement c4-a isoform 1 | XP_002929679 | 0 | 95 | 730.7 | 383 |
| AgU001920_v1.1 | 1944 | 5 | tapasin-related protein | XP_002922294 | 0 | 94 | 728.4 | 431 |
| AgU001981_v1.1 | 1931 | 0 | protein red | NP_001096819 | 0 | 99 | 854.0 | 555 |
| AgU001997_v1.1 | 1926 | 2 | semaphorin-3f precursor | AES06272 | 0 | 98 | 1128.6 | 603 |
| AgU002003_v1.1 | 1922 | 0 | cathepsin c | XP_002925058 | 0 | 95 | 792.7 | 404 |
| AgU002046_v1.1 | 1899 | 0 | phosphatidylinositol 3-kinase regulatory subunit gamma | XP_002715177 | 0 | 90 | 836.6 | 477 |
| AgU002092_v1.1 | 1892 | 1 | protein orai-3 | XP_849021 | 9.22E-180 | 96 | 522.7 | 296 |
| AgU002154_v1.1 | 1868 | 1 | interleukin-4 receptor alfa chain | XP_002929252 | 5.53E-109 | 84 | 357.5 | 285 |
| AgU002198_v1.1 | 1857 | 1 | proteasome maturation protein | XP_003363225 | 8.89E-082 | 92 | 265.8 | 162 |
| AgU002268_v1.1 | 1833 | 0 | ccaat enhancer-binding protein beta | XP_003639913 | 6.24E-119 | 100 | 368.2 | 286 |
| AgU002291_v1.1 | 1828 | 2 | complement c1r subcomponent precursor | XP_002922265 | 0 | 98 | 1184.9 | 583 |
| AgU002340_v1.1 | 1812 | 0 | transcription factor 4 | XP_003406339 | 0 | 100 | 777.3 | 488 |
| AgU002354_v1.1 | 1811 | 2 | ankyrin repeat family a protein 2 | XP_002929568 | 0 | 100 | 650.6 | 313 |
| AgU002372_v1.1 | 1803 | 0 | atlastin-2 isoform 2 | XP_003639637 | 5.17E-123 | 100 | 319.3 | 151 |
| AgU002397_v1.1 | 1795 | 0 | vav 3 guanine nucleotide exchange factor | XP_003267913 | 4.90E-172 | 96 | 501.5 | 265 |
| AgU002399_v1.1 | 1795 | 3 | plasma protease c1 inhibitor isoform 2 | XP_002922754 | 0 | 92 | 809.3 | 463 |
| AgU002451_v1.1 | 1777 | 1 | complement factor i | XP_002929384 | 0 | 94 | 749.6 | 424 |
| AgU002457_v1.1 | 1775 | 6 | myeloid cell nuclear differentiation antigen | XP_002927318 | 0 | 90 | 632.1 | 388 |
| AgU002491_v1.1 | 1768 | 0 | toll-like receptor 4 | XP_002929935 | 0 | 97 | 768.8 | 412 |
| AgU002508_v1.1 | 1762 | 1 | yth domain family protein 2 | XP_535336 | 0 | 99 | 571.6 | 311 |
| AgU002585_v1.1 | 1740 | 0 | serine threonine-protein kinase tbk1 | AES07902 | 0 | 97 | 447.6 | 222 |
| AgU002639_v1.1 | 1722 | 0 | semaphorin-3c precursor | AES06266 | 3.17E-145 | 100 | 348.6 | 167 |
| AgU002683_v1.1 | 1717 | 0 | dead (asp-glu-ala-asp) box polypeptide 58 | AER97092 | 0 | 97 | 581.3 | 302 |
| AgU002820_v1.1 | 1684 | 2 | interferon-induced protein with tetratricopeptide repeats 3 | XP_848320 | 8.64E-167 | 83 | 318.2 | 233 |
| AgU002944_v1.1 | 1646 | 0 | toll interacting protein | XP_540778 | 5.74E-156 | 97 | 458.0 | 274 |
| AgU002970_v1.1 | 1644 | 0 | gtp-binding protein gem | XP_001927011 | 5.47E-124 | 100 | 377.1 | 183 |
| AgU002977_v1.1 | 1641 | 18 | complement factor h | XP_002929497 | 0 | 91 | 960.7 | 546 |
| AgU002984_v1.1 | 1640 | 0 | interleukin-1 receptor antagonist protein precursor | NP_001003096 | 1.52E-114 | 96 | 348.2 | 176 |
| AgU003041_v1.1 | 1628 | 0 | cd83 antigen | EFB20933 | 3.52E-094 | 90 | 296.2 | 183 |
| AgU003046_v1.1 | 1623 | 4 | 2 -5 -oligoadenylate synthase 1 | NP_001041596 | 0 | 89 | 557.0 | 346 |
| AgU003140_v1.1 | 1608 | 1 | t-cell surface glycoprotein cd1a-like | XP_853335 | 3.38E-173 | 90 | 505.0 | 342 |
| AgU003163_v1.1 | 1597 | 0 | v-type proton atpase 116 kda subunit a isoform 2 | XP_002913184 | 7.35E-097 | 96 | 269.6 | 140 |
| AgU003173_v1.1 | 1590 | 1 | nuclear body protein sp140 | XP_852450 | 0 | 75 | 546.2 | 445 |
| AgU003186_v1.1 | 1593 | 0 | ccaat enhancer-binding protein gamma | AER96033 | 1.18E-069 | 96 | 182.6 | 100 |
| AgU003233_v1.1 | 1586 | 1 | tyrosine-protein kinase syk | XP_002919153 | 0 | 99 | 727.2 | 352 |
| AgU003302_v1.1 | 1570 | 0 | tumor necrosis factor member 13 | NP_001192098 | 8.81E-150 | 97 | 440.3 | 250 |
| AgU003328_v1.1 | 1552 | 0 | interleukin 10 beta | XP_002919634 | 0 | 92 | 649.8 | 375 |
| AgU003449_v1.1 | 1538 | 2 | receptor n large subunit p51 precursor | XP_002917885 | 0 | 96 | 609.0 | 332 |
| AgU003482_v1.1 | 1533 | 1 | tissue factor | AER98217 | 3.83E-104 | 90 | 323.6 | 222 |
| AgU003521_v1.1 | 1527 | 1 | interleukin 8 | NP_999032 | 3.72E-045 | 98 | 163.7 | 100 |
| AgU003551_v1.1 | 1519 | 4 | cd14 antigen | XP_002912663 | 0 | 94 | 666.0 | 355 |
| AgU003552_v1.1 | 1521 | 0 | sam domain and hd domain-containing protein 1 | XP_002915216 | 0 | 99 | 500.7 | 242 |
| AgU003558_v1.1 | 1517 | 2 | chemokine (c-x-c motif) ligand 5 | XP_002919187 | 7.15E-038 | 93 | 144.4 | 100 |
| AgU003613_v1.1 | 1509 | 0 | zinc finger e-box binding homeobox 1 | ABF57341 | 0 | 98 | 878.2 | 494 |
| AgU003731_v1.1 | 1486 | 0 | interleukin-1 beta | Q6R2X3 | 0 | 100 | 557.8 | 270 |
| AgU003794_v1.1 | 1471 | 1 | exosome complex component rrp45 isoform 2 | XP_001154164 | 0 | 96 | 610.1 | 346 |
| AgU003808_v1.1 | 1468 | 0 | c5a anaphylatoxin chemotactic receptor | XP_002923087 | 1.38E-050 | 94 | 186.0 | 119 |
| AgU003835_v1.1 | 1467 | 0 | cathepsin s | EFB28474 | 0 | 94 | 541.2 | 303 |
| AgU003843_v1.1 | 1465 | 0 | b-cell linker protein | XP_002917037 | 0 | 94 | 649.8 | 400 |
| AgU003846_v1.1 | 1465 | 0 | protein cornichon homolog | BAE32615 | 4.33E-074 | 100 | 241.1 | 144 |
| AgU003923_v1.1 | 1454 | 0 | b-cell lymphoma 6 protein | NP_001182333 | 0 | 99 | 815.5 | 395 |
| AgU004117_v1.1 | 1410 | 0 | indoleamine -dioxygenase 1 | XP_002918587 | 0 | 96 | 685.6 | 387 |
| AgU004284_v1.1 | 1389 | 0 | gtp-binding protein rad | XP_003262916 | 1.63E-162 | 96 | 472.6 | 308 |
| AgU004353_v1.1 | 1377 | 0 | cysteine-rich secretory protein 2 precursor | XP_002915473 | 3.55E-155 | 96 | 451.4 | 228 |
| AgU004513_v1.1 | 1351 | 0 | mucosal addressin cell adhesion molecule 1 precursor | XP_002923548 | 5.80E-175 | 91 | 506.9 | 351 |
| AgU004698_v1.1 | 1327 | 0 | complement factor h | XP_002929497 | 1.15E-026 | 57 | 122.5 | 149 |
| AgU004725_v1.1 | 1321 | 2 | beta-galactoside alpha- -sialyltransferase 1 | XP_535839 | 0 | 100 | 611.3 | 288 |
| AgU004763_v1.1 | 1316 | 0 | interleukin 4 receptor | XP_002929252 | 8.82E-155 | 94 | 469.5 | 259 |
| AgU004835_v1.1 | 1305 | 0 | complement factor properdin | XP_002917843 | 0 | 94 | 456.1 | 290 |
| AgU004962_v1.1 | 1292 | 0 | interleukin-6 receptor subunit beta isoform 2 | XP_002927047 | 0 | 100 | 652.5 | 325 |
| AgU004989_v1.1 | 1283 | 0 | sh2b adapter protein 1 isoform 2 | EDL17369 | 1.57E-105 | 86 | 322.0 | 207 |
| AgU005005_v1.1 | 1281 | 2 | natural cytotoxicity triggering receptor 3 ligand 1 | EFB21226 | 4.06E-094 | 88 | 293.9 | 170 |
| AgU005051_v1.1 | 1276 | 0 | fc fragment of high affinity receptor | XP_002931033 | 0 | 93 | 634.0 | 363 |
| AgU005242_v1.1 | 1253 | 0 | tyrosine-protein kinase zap-70 | XP_003431545 | 6.57E-020 | 100 | 100.5 | 49 |
| AgU005277_v1.1 | 1251 | 0 | cd8a molecule | NP_001002935 | 7.33E-016 | 86 | 77.4 | 46 |
| AgU005375_v1.1 | 1238 | 0 | x-box-binding protein 1 | EFB25740 | 1.28E-096 | 97 | 302.4 | 209 |
| AgU005479_v1.1 | 1222 | 0 | transporter associated with antigen presentation partial | AES07826 | 1.22E-111 | 96 | 336.7 | 189 |
| AgU005572_v1.1 | 1213 | 0 | b-cell antigen receptor complex-associated protein beta chain-like isoform 1 | XP_002924557 | 1.41E-112 | 91 | 340.1 | 214 |
| AgU005648_v1.1 | 1208 | 0 | poliovirus receptor-related protein 2 | AES05063 | 0 | 97 | 620.2 | 323 |
| AgU005713_v1.1 | 1199 | 11 | mhc class ii antigen | AAM89225 | 3.12E-156 | 99 | 452.2 | 255 |
| AgU005868_v1.1 | 1179 | 2 | toll-like receptor 2 | AES08217 | 0 | 95 | 589.0 | 325 |
| AgU005899_v1.1 | 1176 | 0 | notch gene homolog 1 | AES03025 | 0 | 97 | 710.3 | 376 |
| AgU005924_v1.1 | 1174 | 4 | immunoglobulin j chain | EFB23253 | 3.15E-028 | 96 | 116.7 | 58 |
| AgU005959_v1.1 | 1169 | 3 | rho gdp-dissociation inhibitor 2 | XP_002928425 | 2.42E-105 | 100 | 224.9 | 107 |
| AgU006060_v1.1 | 1159 | 0 | v-ets erythroblastosis virus e26 oncogene-like 1 | BAG62940 | 0 | 99 | 550.1 | 266 |
| AgU006076_v1.1 | 1155 | 0 | interferon-induced helicase c domain-containing protein 1 | XP_002924913 | 0 | 99 | 577.0 | 276 |
| AgU006102_v1.1 | 1150 | 0 | semaphorin-3c precursor | XP_003357538 | 0 | 100 | 795.0 | 382 |
| AgU006268_v1.1 | 1133 | 0 | interleukin-1 receptor-associated kinase 1 | XP_002928869 | 1.43E-017 | 92 | 92.8 | 66 |
| AgU006300_v1.1 | 1130 | 0 | transient receptor potential cation channel subfamily m member 4 | XP_002917956 | 5.87E-130 | 98 | 413.3 | 249 |
| AgU006317_v1.1 | 1129 | 2 | c-x-c motif chemokine 10 precursor | XP_002924776 | 5.48E-054 | 96 | 184.1 | 98 |
| AgU006400_v1.1 | 1119 | 3 | complement component q c chain | XP_002919574 | 2.15E-132 | 96 | 390.2 | 245 |
| AgU006427_v1.1 | 1117 | 1 | c-type lectin domain family 7 member a | XP_003365219 | 7.15E-093 | 87 | 287.3 | 199 |
| AgU006469_v1.1 | 1111 | 1 | interleukin 1 type i | XP_002913539 | 0 | 96 | 441.8 | 230 |
| AgU006508_v1.1 | 1107 | 0 | nuclear pore complex protein nup88 | XP_848977 | 0 | 93 | 580.1 | 360 |
| AgU006524_v1.1 | 1106 | 4 | interleukin-1 receptor type 2 isoform 1 | XP_002913551 | 0 | 96 | 582.4 | 296 |
| AgU006578_v1.1 | 1102 | 0 | prostaglandin e2 receptor ep4 subtype | XP_002922659 | 1.28E-096 | 100 | 307.0 | 166 |
| AgU006617_v1.1 | 1098 | 0 | interleukin 6 | Q28819 | 2.00E-134 | 97 | 393.7 | 206 |
| AgU006665_v1.1 | 1092 | 2 | b-cell receptor-associated protein 31 | XP_002929128 | 1.14E-134 | 96 | 397.5 | 277 |
| AgU006667_v1.1 | 1090 | 0 | complement component 6 | XP_001496433 | 3.26E-177 | 92 | 446.0 | 254 |
| AgU006708_v1.1 | 1090 | 0 | drebrin-like protein isoform 3 | CAH91655 | 4.37E-031 | 98 | 129.0 | 58 |
| AgU006814_v1.1 | 1067 | 1 | proteasome activator complex subunit 1 | XP_002921125 | 4.18E-152 | 100 | 439.5 | 249 |
| AgU006823_v1.1 | 1077 | 8 | btb poz domain-containing adapter for cul3-mediated degradation protein 2 | AES08520 | 0 | 97 | 600.5 | 301 |
| AgU006912_v1.1 | 1069 | 0 | proteasome ( macropain) beta 8 (large multifunctional peptidase 7) | XP_002914391 | 0 | 97 | 545.8 | 276 |
| AgU006925_v1.1 | 1065 | 0 | interleukin-1 alpha precursor | XP_002926377 | 1.70E-140 | 96 | 411.0 | 224 |
| AgU006963_v1.1 | 1058 | 0 | transcription factor 7-like 2 isoform 4 | EDL94481 | 6.48E-033 | 100 | 135.2 | 73 |
| AgU007080_v1.1 | 1052 | 2 | 2 -5 -oligoadenylate synthetase-like | EFB23134 | 4.12E-080 | 90 | 264.2 | 166 |
| AgU007161_v1.1 | 1048 | 0 | cd209 antigen | NP_001124304 | 2.58E-134 | 94 | 394.0 | 238 |
| AgU007232_v1.1 | 1037 | 0 | twinfilin-2 | AES09761 | 0 | 100 | 515.4 | 281 |
| AgU007366_v1.1 | 1032 | 0 | bpi fold-containing family a member 1 precursor | XP_002918312 | 1.29E-098 | 94 | 303.1 | 255 |
| AgU007371_v1.1 | 1032 | 0 | interferon regulatory factor 8 | AFE69981 | 2.09E-046 | 92 | 165.2 | 97 |
| AgU007433_v1.1 | 1027 | 0 | triggering receptor expressed on myeloid cells 1 | XP_002914564 | 1.94E-141 | 94 | 411.8 | 241 |
| AgU007437_v1.1 | 1028 | 1 | complement component q a chain | XP_002919572 | 3.16E-111 | 97 | 335.1 | 245 |
| AgU007480_v1.1 | 1021 | 0 | transforming growth beta 2 | 1TFG_A | 1.11E-042 | 100 | 154.1 | 73 |
| AgU007556_v1.1 | 1018 | 4 | bone marrow stromal cell antigen 2 | XP_002912835 | 7.29E-062 | 81 | 213.0 | 173 |
| AgU007608_v1.1 | 1003 | 0 | serpin b3-like | XP_002926409 | 1.53E-089 | 95 | 196.1 | 108 |
| AgU007657_v1.1 | 1010 | 1 | pou domain class 2-associating factor 1 | AES04487 | 0 | 98 | 520.8 | 281 |
| AgU007748_v1.1 | 1001 | 0 | complement component 2 | XP_002929672 | 0 | 98 | 675.2 | 333 |
| AgU007780_v1.1 | 999 | 0 | tyrosine-protein kinase syk isoform 1 | XP_003432699 | 0 | 98 | 579.3 | 281 |
| AgU007785_v1.1 | 995 | 0 | 54 kda 2 -5 -oligoadenylate synthase-like protein 2-like | EFB23133 | 1.26E-166 | 92 | 485.3 | 295 |
| AgU007841_v1.1 | 994 | 2 | complement c1q subcomponent subunit b | XP_002919576 | 1.06E-110 | 95 | 333.2 | 249 |
| AgU007843_v1.1 | 995 | 0 | mothers against decapentaplegic homolog 3 isoform 2 | BAE00330 | 2.15E-047 | 100 | 169.9 | 79 |
| AgU008000_v1.1 | 983 | 3 | complement factor h | XP_536110 | 3.63E-120 | 81 | 384.8 | 241 |
| AgU008016_v1.1 | 980 | 0 | proto-oncogene vav | EFB15363 | 1.99E-171 | 98 | 484.6 | 242 |
| AgU008104_v1.1 | 974 | 0 | e3 ubiquitin-protein ligase cbl- partial | AFE72312 | 1.52E-011 | 100 | 68.9 | 35 |
| AgU008224_v1.1 | 965 | 7 | signal transducer and activator of transcription 5a | XP_002922177 | 0 | 99 | 582.0 | 305 |
| AgU008279_v1.1 | 961 | 0 | interleukin-18 precursor | XP_002921155 | 5.76E-126 | 97 | 369.8 | 193 |
| AgU008344_v1.1 | 954 | 0 | leukosialin precursor | NP_033285 | 7.04E-038 | 77 | 148.3 | 127 |
| AgU008353_v1.1 | 955 | 1 | c4b-binding protein alpha chain | XP_002928724 | 1.91E-114 | 95 | 340.5 | 198 |
| AgU008400_v1.1 | 954 | 1 | ferritin heavy chain | AAH89817 | 1.93E-137 | 87 | 400.2 | 243 |
| AgU008485_v1.1 | 946 | 1 | chemokine (c-c motif) ligand 13 | AER95719 | 2.85E-046 | 92 | 162.2 | 98 |
| AgU008582_v1.1 | 940 | 0 | ubiquitin thioesterase otub1 | XP_003828597 | 1.93E-147 | 99 | 385.2 | 200 |
| AgU008588_v1.1 | 940 | 2 | membrane cofactor protein | EFB21611 | 8.34E-149 | 88 | 430.3 | 262 |
| AgU008617_v1.1 | 939 | 0 | interferon-induced guanylate-binding protein 1 | AER98962 | 6.02E-155 | 95 | 458.0 | 273 |
| AgU008762_v1.1 | 927 | 1 | hla class ii histocompatibility dm beta chain-like | XP_002914389 | 3.58E-081 | 93 | 257.7 | 150 |
| AgU008889_v1.1 | 921 | 0 | preli domain containing 1 | XP_003806905 | 1.49E-107 | 98 | 323.2 | 189 |
| AgU009024_v1.1 | 912 | 0 | semaphorin-7a isoform 2 | XP_002923040 | 1.76E-143 | 98 | 428.7 | 209 |
| AgU009047_v1.1 | 910 | 0 | complement component gamma polypeptide | EFB23711 | 6.58E-099 | 91 | 300.4 | 198 |
| AgU009052_v1.1 | 908 | 0 | c-c chemokine receptor type 11 | XP_002926295 | 2.13E-079 | 99 | 255.8 | 135 |
| AgU009079_v1.1 | 909 | 0 | toll-like receptor 6 | XP_002928171 | 1.31E-177 | 97 | 522.3 | 268 |
| AgU009124_v1.1 | 908 | 0 | dna-binding protein rfxank isoform 1 | EFB19142 | 1.86E-149 | 98 | 430.3 | 236 |
| AgU009160_v1.1 | 905 | 1 | proteasome subunit beta type-9 | AAC50154 | 5.98E-092 | 97 | 282.7 | 209 |
| AgU009169_v1.1 | 903 | 0 | interferon-induced protein with tetratricopeptide repeats 2 | XP_002914449 | 2.81E-111 | 83 | 311.2 | 216 |
| AgU009236_v1.1 | 897 | 1 | proteasome activator complex subunit 2 | XP_002921123 | 6.70E-125 | 98 | 349.4 | 173 |
| AgU009288_v1.1 | 893 | 2 | interleukin-27 receptor subunit alpha | EFB28637 | 1.16E-152 | 86 | 452.2 | 283 |
| AgU009517_v1.1 | 878 | 0 | phospholipid scramblase 3 | EHB04477 | 3.07E-038 | 100 | 112.1 | 55 |
| AgU009589_v1.1 | 874 | 0 | complement component 2 | XP_002929674 | 0 | 98 | 596.7 | 290 |
| AgU009761_v1.1 | 865 | 0 | caspase recruitment domain-containing protein 11 | XP_547005 | 3.66E-100 | 98 | 328.2 | 182 |
| AgU009764_v1.1 | 865 | 0 | c-c motif chemokine 21 precursor | XP_002930570 | 3.77E-049 | 92 | 169.9 | 105 |
| AgU009811_v1.1 | 857 | 0 | zinc-alpha-2-glycoprotein precursor | XP_002930457 | 1.40E-116 | 95 | 349.0 | 191 |
| AgU009999_v1.1 | 853 | 2 | clusterin precursor | AER96420 | 4.15E-114 | 98 | 339.0 | 197 |
| AgU010008_v1.1 | 850 | 0 | signal transducer and activator of transcription 5a | XP_003362605 | 2.13E-079 | 99 | 265.8 | 138 |
| AgU010043_v1.1 | 848 | 0 | phosphoprotein associated with glycosphingolipid-enriched microdomains 1 | AES03484 | 5.28E-092 | 95 | 285.8 | 180 |
| AgU010098_v1.1 | 847 | 0 | neural wiskott-aldrich syndrome protein | XP_003896577 | 0 | 97 | 529.6 | 281 |
| AgU010117_v1.1 | 845 | 0 | complement component (3d epstein barr virus) receptor 2 | XP_002928718 | 1.98E-142 | 96 | 439.1 | 245 |
| AgU010205_v1.1 | 841 | 7 | mhc class ii antigen | AAS45656 | 1.16E-085 | 100 | 268.1 | 151 |
| AgU010239_v1.1 | 839 | 0 | cd1e molecule | XP_545727 | 2.73E-146 | 85 | 427.2 | 278 |
| AgU010312_v1.1 | 836 | 4 | complement component q subcomponent binding protein | XP_001918153 | 1.45E-121 | 98 | 360.1 | 192 |
| AgU010364_v1.1 | 831 | 0 | complement component c6 | XP_536488 | 2.55E-135 | 95 | 380.9 | 202 |
| AgU010383_v1.1 | 830 | 0 | ubiquitin thioesterase otub2 | AES03419 | 2.63E-164 | 99 | 466.8 | 226 |
| AgU010402_v1.1 | 830 | 0 | b-cell cll lymphoma 6 member b protein | XP_546577 | 1.51E-139 | 98 | 412.9 | 205 |
| AgU010466_v1.1 | 827 | 0 | complement factor h | EFB19334 | 1.09E-129 | 97 | 336.7 | 171 |
| AgU010559_v1.1 | 821 | 0 | c-c motif chemokine 20 | AFQ90557 | 3.08E-045 | 95 | 157.9 | 96 |
| AgU010659_v1.1 | 818 | 3 | interferon alpha-inducible protein 6 isoform a | XP_535344 | 1.26E-029 | 83 | 118.6 | 130 |
| AgU010717_v1.1 | 811 | 0 | antigen peptide transporter 2 | EFB14066 | 3.87E-073 | 94 | 246.9 | 159 |
| AgU010789_v1.1 | 807 | 0 | vitronectin | AES09612 | 2.99E-139 | 98 | 411.8 | 241 |
| AgU010828_v1.1 | 808 | 0 | tyrosine-protein kinase zap-70 | XP_002912473 | 1.54E-146 | 97 | 435.3 | 214 |
| AgU010994_v1.1 | 791 | 0 | secreted and transmembrane 1 | EFB20412 | 8.94E-054 | 69 | 179.5 | 151 |
| AgU011272_v1.1 | 782 | 0 | interferon-induced protein with tetratricopeptide repeats 1-like | EFB20310 | 2.83E-150 | 91 | 439.1 | 260 |
| AgU011362_v1.1 | 780 | 0 | cd74 major histocompatibility class ii invariant chain | XP_002918205 | 1.93E-108 | 96 | 327.0 | 192 |
| AgU011425_v1.1 | 777 | 0 | drebrin-like protein isoform b | AER96985 | 5.57E-126 | 97 | 374.0 | 252 |
| AgU011439_v1.1 | 777 | 0 | t-cell surface glycoprotein cd4 precursor | EFB13967 | 6.91E-075 | 92 | 225.7 | 145 |
| AgU011516_v1.1 | 772 | 0 | interferon regulatory factor 7 | XP_002929528 | 3.46E-112 | 96 | 342.0 | 216 |
| AgU011545_v1.1 | 769 | 0 | interleukin-1 receptor accessory protein | EFB27553 | 1.73E-084 | 88 | 273.1 | 158 |
| AgU011552_v1.1 | 772 | 1 | chemokine (c-c motif) ligand 2 | XP_002912400 | 4.22E-051 | 96 | 172.9 | 101 |
| AgU011557_v1.1 | 771 | 0 | sh2b adapter protein 3 | XP_003433461 | 1.13E-179 | 97 | 518.5 | 257 |
| AgU011682_v1.1 | 764 | 0 | sorting nexin 7 | XP_547269 | 5.25E-109 | 98 | 330.9 | 164 |
| AgU011726_v1.1 | 759 | 0 | hla class ii histocompatibility do beta chain-like | XP_002914393 | 1.47E-159 | 96 | 455.3 | 232 |
| AgU011756_v1.1 | 761 | 2 | interleukin-36 beta | XP_002926386 | 4.53E-073 | 95 | 231.1 | 116 |
| AgU011800_v1.1 | 759 | 0 | 54 kda 2 -5 -oligoadenylate synthase-like protein 2-like | NP_001041558 | 5.23E-064 | 89 | 218.4 | 131 |
| AgU011860_v1.1 | 755 | 0 | tumor necrosis factor receptor member 14 (herpesvirus entry mediator) | XP_549666 | 1.25E-103 | 83 | 313.2 | 227 |
| AgU011873_v1.1 | 755 | 0 | c-c motif chemokine 27 | XP_002930569 | 4.38E-041 | 97 | 147.1 | 88 |
| AgU012091_v1.1 | 746 | 0 | gamma-interferon-inducible lysosomal thiol reductase-like | EFB19113 | 1.34E-111 | 97 | 330.5 | 171 |
| AgU012283_v1.1 | 737 | 0 | cd6 molecule | EFB23063 | 5.78E-136 | 95 | 407.1 | 245 |
| AgU012318_v1.1 | 736 | 3 | chemokine (c-x-c motif) ligand 14 | XP_002913013 | 3.76E-053 | 100 | 171.8 | 81 |
| AgU012445_v1.1 | 728 | 0 | protein c-ets-1 | 3MFK_A | 7.32E-083 | 86 | 255.8 | 154 |
| AgU012599_v1.1 | 721 | 0 | interleukin-1 receptor-associated kinase 1 | XP_002928869 | 4.04E-139 | 96 | 416.8 | 240 |
| AgU012869_v1.1 | 711 | 1 | c-c motif chemokine 3-like 1-like | AES11085 | 2.96E-042 | 88 | 149.1 | 89 |
| AgU012889_v1.1 | 708 | 0 | linker for activation of t-cells family member 1 | XP_002927386 | 2.85E-044 | 95 | 153.7 | 84 |
| AgU012925_v1.1 | 710 | 1 | cd55 decay accelerating factor for complement (cromer blood group) | XP_002928720 | 3.32E-090 | 82 | 247.3 | 179 |
| AgU013076_v1.1 | 704 | 0 | interferon-induced guanylate-binding protein 1 | EFB14092 | 3.71E-160 | 97 | 466.5 | 230 |
| AgU013094_v1.1 | 702 | 0 | lymphocyte antigen 86 | XP_535877 | 1.59E-083 | 94 | 256.9 | 134 |
| AgU013140_v1.1 | 699 | 0 | antigen peptide transporter 2 | EFB14066 | 9.95E-079 | 94 | 260.4 | 153 |
| AgU013159_v1.1 | 700 | 0 | phosphatidylinositol 3-kinase regulatory subunit alpha isoform 2 | XP_003433863 | 7.64E-145 | 99 | 422.6 | 204 |
| AgU013173_v1.1 | 699 | 0 | chemokine (c-x3-c motif) ligand 1 | EFB22226 | 1.21E-061 | 94 | 208.0 | 147 |
| AgU013216_v1.1 | 698 | 0 | mitochondrial antiviral-signaling protein | XP_002918808 | 2.27E-105 | 88 | 325.1 | 208 |
| AgU013299_v1.1 | 694 | 0 | e3 ubiquitin-protein ligase rnf125 | XP_547620 | 1.85E-092 | 98 | 282.3 | 140 |
| AgU013340_v1.1 | 692 | 0 | c-c motif chemokine 19-like | XP_002930573 | 5.97E-052 | 98 | 174.1 | 84 |
| AgU013354_v1.1 | 694 | 0 | atlastin-2 isoform 1 | XP_002914883 | 1.98E-158 | 100 | 462.6 | 220 |
| AgU013408_v1.1 | 689 | 1 | interleukin-1 family member 10 | XP_854689 | 2.64E-033 | 94 | 127.5 | 67 |
| AgU013494_v1.1 | 687 | 0 | granulocyte colony-stimulating factor receptor | XP_002925207 | 2.32E-108 | 92 | 341.3 | 192 |
| AgU013517_v1.1 | 684 | 0 | adenosine deaminase | EFB21151 | 2.21E-151 | 95 | 435.6 | 227 |
| AgU013716_v1.1 | 678 | 0 | serpin b13 | XP_002917546 | 9.90E-124 | 95 | 367.5 | 204 |
| AgU013769_v1.1 | 676 | 0 | zinc finger e-box binding homeobox 1 | AES10074 | 4.47E-140 | 97 | 422.9 | 225 |
| AgU013785_v1.1 | 674 | 1 | complement component r subcomponent | XP_002922265 | 2.44E-079 | 100 | 261.9 | 120 |
| AgU013794_v1.1 | 673 | 0 | apolipoprotein b editing enzyme catalytic polypeptide-like 3c | ABV71926 | 9.00E-086 | 79 | 263.5 | 191 |
| AgU013802_v1.1 | 675 | 0 | tripartite motif-containing protein 34 | NP_001192120 | 5.08E-045 | 93 | 166.4 | 82 |
| AgU013963_v1.1 | 668 | 0 | interferon gamma receptor 2 | XP_002919631 | 1.84E-139 | 94 | 409.1 | 221 |
| AgU014051_v1.1 | 665 | 0 | interferon-stimulated transcription factor gamma 48kda | EFB17596 | 8.23E-056 | 97 | 193.0 | 93 |
| AgU014107_v1.1 | 660 | 0 | p2y purinoceptor 14 | AES03449 | 6.25E-067 | 97 | 219.9 | 112 |
| AgU014218_v1.1 | 655 | 0 | fyn-binding protein | AER98831 | 2.20E-076 | 96 | 239.6 | 158 |
| AgU014292_v1.1 | 655 | 1 | high affinity immunoglobulin epsilon receptor subunit gamma precursor | NP_001124473 | 4.33E-045 | 93 | 155.6 | 86 |
| AgU014349_v1.1 | 652 | 1 | interleukin 1 member 9 | XP_002926387 | 3.27E-084 | 74 | 260.0 | 215 |
| AgU014369_v1.1 | 650 | 0 | complement c5 | EFB13296 | 1.16E-115 | 97 | 370.9 | 194 |
| AgU014376_v1.1 | 652 | 0 | semaphorin-7a isoform 2 | XP_002923040 | 3.89E-153 | 98 | 449.9 | 217 |
| AgU014378_v1.1 | 652 | 0 | b-cell lymphoma 6 protein | XP_003792760 | 3.36E-060 | 99 | 209.9 | 100 |
| AgU014570_v1.1 | 644 | 0 | b-cell lymphoma 6 protein | AER95017 | 3.64E-145 | 98 | 424.1 | 214 |
| AgU014593_v1.1 | 642 | 0 | zinc finger e-box-binding homeobox 2 isoform 1 | AES10075 | 6.98E-156 | 99 | 451.1 | 213 |
| AgU014603_v1.1 | 642 | 0 | tnf receptor-associated factor 6 | XP_002926543 | 7.55E-142 | 100 | 418.3 | 213 |
| AgU014620_v1.1 | 642 | 0 | mitogen-activated protein kinase kinase kinase kinase 2 | ACC64576 | 1.61E-131 | 99 | 400.6 | 213 |
| AgU014686_v1.1 | 639 | 0 | interferon-stimulated protein 17 ubiquitin cross-reactive protein | XP_002930209 | 1.54E-093 | 92 | 282.3 | 165 |
| AgU014744_v1.1 | 637 | 0 | mhc class ii transactivator | XP_003481094 | 8.95E-018 | 100 | 87.0 | 40 |
| AgU014751_v1.1 | 637 | 0 | wiskott-aldrich syndrome protein | AES09632 | 5.78E-082 | 97 | 260.0 | 173 |
| AgU014863_v1.1 | 634 | 0 | t-cell receptor beta chain t17t-22-like | XP_002924221 | 1.19E-120 | 95 | 356.3 | 188 |
| AgU015034_v1.1 | 628 | 0 | chemokine (c-c motif) ligand 9 | XP_002923673 | 3.00E-054 | 87 | 179.9 | 108 |
| AgU015077_v1.1 | 624 | 0 | chromogranin a | XP_002923879 | 1.27E-091 | 94 | 286.6 | 154 |
| AgU015267_v1.1 | 617 | 5 | deleted in malignant brain tumors 1 | BAG65024 | 5.05E-094 | 84 | 290.8 | 186 |
| AgU015312_v1.1 | 617 | 0 | chromogranin a (parathyroid secretory protein 1) | EHH28122 | 1.39E-050 | 94 | 179.9 | 119 |
| AgU015339_v1.1 | 616 | 0 | lymphocyte antigen 96 | XP_002930732 | 4.49E-104 | 95 | 308.1 | 160 |
| AgU015702_v1.1 | 598 | 2 | ferritin heavy chain-like | XP_002917628 | 4.08E-076 | 87 | 237.3 | 141 |
| AgU015706_v1.1 | 597 | 0 | leukocyte specific transcript 1 | XP_002930077 | 4.13E-032 | 82 | 122.5 | 105 |
| AgU015720_v1.1 | 602 | 0 | interleukin-1 receptor-associated kinase 3 | XP_538271 | 1.98E-104 | 96 | 324.3 | 176 |
| AgU015762_v1.1 | 601 | 0 | c-c chemokine receptor type 1 | EFB19170 | 8.10E-107 | 95 | 322.0 | 198 |
| AgU015978_v1.1 | 591 | 1 | immunoglobulin-like transcript 8-like | XP_001491661 | 8.34E-034 | 77 | 110.9 | 80 |
| AgU016010_v1.1 | 590 | 0 | e3 ubiquitin-protein ligase march8 | XP_002926290 | 4.62E-143 | 100 | 411.4 | 196 |
| AgU016024_v1.1 | 593 | 0 | neurogenic locus notch homolog protein 2 | AES03026 | 3.98E-133 | 100 | 401.7 | 197 |
| AgU016077_v1.1 | 591 | 0 | inhibitor of nuclear factor kappa-b kinase subunit epsilon isoform 1 | AES00134 | 2.10E-136 | 99 | 399.8 | 195 |
| AgU016286_v1.1 | 586 | 0 | c-c motif chemokine 3 precursor | AER95730 | 1.34E-046 | 96 | 158.7 | 93 |
| AgU016326_v1.1 | 582 | 0 | platelet factor 4-like | AES11338 | 1.48E-032 | 86 | 122.9 | 90 |
| AgU016364_v1.1 | 582 | 0 | semaphorin-4d | XP_002742761 | 2.74E-046 | 67 | 141.7 | 151 |
| AgU016419_v1.1 | 580 | 0 | tumor necrosis factor receptor superfamily member 6 | AER98401 | 1.33E-074 | 86 | 235.3 | 152 |
| AgU016432_v1.1 | 580 | 2 | clusterin precursor | XP_002914459 | 3.23E-085 | 97 | 269.2 | 138 |
| AgU016595_v1.1 | 574 | 2 | interferon regulatory factor 4 | XP_002930176 | 1.60E-093 | 100 | 283.5 | 134 |
| AgU016617_v1.1 | 573 | 0 | aquaporin-9 | XP_002920714 | 3.02E-095 | 97 | 290.0 | 191 |
| AgU016647_v1.1 | 571 | 0 | fas apoptotic inhibitory molecule 3 | AER98244 | 1.77E-063 | 91 | 211.8 | 170 |
| AgU016768_v1.1 | 568 | 0 | major histocompatibility class dm alpha | CAI11403 | 1.94E-097 | 94 | 294.3 | 169 |
| AgU016837_v1.1 | 563 | 0 | aquaporin-9 | XP_544701 | 4.65E-029 | 96 | 118.6 | 60 |
| AgU016841_v1.1 | 564 | 0 | g-protein coupled receptor 183 | EFB20780 | 6.12E-035 | 100 | 135.6 | 78 |
| AgU016904_v1.1 | 563 | 0 | src kinase-associated phosphoprotein 1 | XP_537665 | 4.06E-111 | 99 | 332.4 | 186 |
| AgU017043_v1.1 | 557 | 13 | immunoglobulin heavy chain variable region subgroup 3 | AAO17823 | 5.59E-047 | 76 | 116.3 | 85 |
| AgU017077_v1.1 | 560 | 2 | mhc class ii antigen | AAS45653 | 5.59E-134 | 100 | 386.3 | 186 |
| AgU017125_v1.1 | 557 | 0 | granzyme a | AER99536 | 2.83E-079 | 94 | 247.7 | 127 |
| AgU017445_v1.1 | 549 | 0 | immunoglobulin mu heavy chain | AAX73309 | 3.54E-081 | 97 | 258.8 | 130 |
| AgU017464_v1.1 | 546 | 0 | cd276 antigen isoform 2 | XP_001925680 | 4.43E-126 | 99 | 368.2 | 181 |
| AgU017469_v1.1 | 547 | 1 | tumor necrosis factor ligand superfamily member 12 | AES08546 | 5.98E-099 | 98 | 296.2 | 165 |
| AgU017541_v1.1 | 545 | 0 | interleukin-6 receptor subunit beta | EFB18858 | 1.10E-121 | 99 | 375.6 | 181 |
| AgU017575_v1.1 | 539 | 0 | b-cell cll lymphoma 2 | AFE72075 | 4.48E-076 | 99 | 235.0 | 124 |
| AgU017610_v1.1 | 542 | 0 | interferon-induced protein 35 | EFB24208 | 2.40E-075 | 93 | 238.0 | 132 |
| AgU017730_v1.1 | 539 | 0 | programmed cell death 1 | EFB16570 | 3.17E-085 | 93 | 262.7 | 179 |
| AgU017813_v1.1 | 537 | 0 | major histocompatibility class dm beta | NP_001257999 | 2.01E-054 | 93 | 183.7 | 95 |
| AgU017875_v1.1 | 535 | 7 | ig heavy chain v-iii region vh26-like | XP_003778445 | 1.16E-073 | 82 | 233.8 | 151 |
| AgU017910_v1.1 | 536 | 0 | cytokine receptor common subunit gamma precursor | AES00201 | 1.51E-060 | 97 | 203.0 | 121 |
| AgU018018_v1.1 | 531 | 0 | x-box-binding protein 1 | NP_001029899 | 3.05E-057 | 88 | 191.0 | 165 |
| AgU018046_v1.1 | 530 | 0 | adenosine deaminase | XP_534428 | 1.62E-043 | 95 | 157.9 | 81 |
| AgU018217_v1.1 | 525 | 0 | nf-kappa-b essential modulator | EAW72679 | 2.52E-073 | 97 | 232.6 | 147 |
| AgU018395_v1.1 | 515 | 11 | immunogobulin gamma 1 heavy chain constant region | AES00113 | 3.32E-038 | 95 | 136.0 | 70 |
| AgU018450_v1.1 | 518 | 1 | toll-like receptor 2 | AES08216 | 4.13E-069 | 89 | 219.6 | 148 |
| AgU018498_v1.1 | 515 | 0 | fyn-binding protein | AER98832 | 3.30E-029 | 100 | 114.4 | 55 |
| AgU018723_v1.1 | 512 | 0 | sema immunoglobulin domain transmembrane domain and short cytoplasmic 4d | AES06277 | 3.48E-032 | 96 | 131.0 | 62 |
| AgU018860_v1.1 | 505 | 22 | immunoglobulin heavy chain variable region | CAD60332 | 2.34E-054 | 83 | 178.7 | 112 |
| AgU018936_v1.1 | 506 | 16 | ig heavy chain v-iii region vh26-like | AAA69729 | 1.99E-073 | 83 | 228.4 | 153 |
| AgU019021_v1.1 | 504 | 0 | interleukin-1 receptor-associated kinase 1 isoform 1 | XP_549367 | 3.33E-054 | 100 | 191.8 | 88 |
| AgU019083_v1.1 | 500 | 0 | gtp-binding protein 1 | EGW01685 | 1.06E-117 | 100 | 346.7 | 166 |
| AgU019143_v1.1 | 499 | 22 | immunoglobulin lambda light chain | S25738 | 3.42E-079 | 83 | 245.7 | 159 |
| AgU019172_v1.1 | 495 | 13 | immunoglobulin heavy chain | AAL35301 | 4.25E-039 | 66 | 147.5 | 159 |
| AgU019177_v1.1 | 496 | 2 | ig heavy chain v-iii region vh26-like | XP_510217 | 5.03E-069 | 83 | 217.6 | 139 |
| AgU019192_v1.1 | 498 | 18 | immunoglobulin kappa light chain v-j region | ADK09517 | 2.09E-065 | 83 | 208.8 | 136 |
| AgU019201_v1.1 | 496 | 1 | interferon-induced protein 44-like | EFB17672 | 2.11E-041 | 93 | 153.3 | 81 |
| AgU019233_v1.1 | 498 | 0 | transcription factor 4 isoform 1 | NP_003190 | 3.15E-109 | 99 | 336.7 | 166 |
| AgU019478_v1.1 | 489 | 0 | lymphotoxin-beta | AES01364 | 9.84E-099 | 98 | 295.8 | 162 |
| AgU019690_v1.1 | 486 | 0 | interferon-induced protein with tetratricopeptide repeats 1 | EFB20310 | 2.93E-044 | 97 | 161.4 | 85 |
| AgU019738_v1.1 | 483 | 0 | interleukin-6 receptor subunit beta isoform 2 | XP_003782803 | 1.24E-073 | 97 | 202.2 | 116 |
| AgU020009_v1.1 | 480 | 0 | cd27 antigen | XP_854464 | 4.33E-067 | 95 | 215.7 | 110 |
| AgU020036_v1.1 | 477 | 9 | ig heavy chain v-iii region vh26-like | AAA69729 | 2.33E-072 | 78 | 225.3 | 156 |
| AgU020311_v1.1 | 456 | 25 | ig heavy chain v-iii region vh26-like | XP_001149684 | 2.27E-056 | 73 | 184.5 | 135 |
| AgU020336_v1.1 | 469 | 0 | cd8 alpha chain | ABS50091 | 4.19E-063 | 90 | 204.5 | 140 |
| AgU020459_v1.1 | 465 | 0 | transcription factor 12 | NP_001071353 | 7.40E-099 | 100 | 309.3 | 154 |
| AgU020464_v1.1 | 465 | 0 | src-like-adaptor | XP_002917660 | 7.79E-107 | 100 | 318.5 | 154 |
| AgU020487_v1.1 | 464 | 3 | immunoglobulin heavy chain variable region subgroup 3 | AAA69729 | 6.58E-051 | 78 | 147.5 | 104 |
| AgU020520_v1.1 | 465 | 0 | granulocyte colony-stimulating factor | XP_850306 | 2.55E-075 | 90 | 236.5 | 155 |
| AgU020547_v1.1 | 462 | 0 | c-x-c motif chemokine 9 precursor | ABS50092 | 5.97E-020 | 89 | 89.4 | 49 |
| AgU020589_v1.1 | 462 | 0 | mhc class ii regulatory factor rfx1 | AFE75547 | 1.51E-049 | 100 | 166.0 | 80 |
| AgU020596_v1.1 | 462 | 0 | interferon regulatory factor 9 | EFB17596 | 2.52E-059 | 92 | 199.1 | 153 |
| AgU020746_v1.1 | 457 | 16 | ig heavy chain v-iii region vh26-like | XP_510217 | 3.54E-060 | 75 | 194.5 | 141 |
| AgU020754_v1.1 | 457 | 0 | major histocompatibility class dm alpha | XP_002914415 | 9.72E-035 | 100 | 131.7 | 81 |
| AgU020878_v1.1 | 452 | 25 | immunoglobulin heavy chain variable region | XP_003778445 | 1.51E-049 | 79 | 131.0 | 89 |
| AgU021010_v1.1 | 450 | 14 | igl@ protein | AAO46065 | 3.28E-061 | 83 | 196.8 | 149 |
| AgU021037_v1.1 | 450 | 0 | cd40 tnf receptor superfamily member 5 | EFB21982 | 1.63E-035 | 91 | 133.3 | 107 |
| AgU021142_v1.1 | 447 | 0 | complement c5 | EFB13296 | 8.20E-048 | 94 | 176.0 | 91 |
| AgU021405_v1.1 | 441 | 0 | immunoglobulin superfamily member 6 | XP_002926106 | 1.69E-086 | 90 | 263.5 | 142 |
| AgU021472_v1.1 | 440 | 9 | ig heavy chain v-iii region vh26-like | XP_003465027 | 3.05E-048 | 85 | 162.5 | 91 |
| AgU021541_v1.1 | 428 | 0 | serine threonine-protein kinase tbk1 | AES07902 | 9.01E-053 | 91 | 186.4 | 96 |
| AgU021570_v1.1 | 436 | 13 | immunoglobulin heavy chain variable region | AAW63077 | 7.33E-067 | 86 | 211.5 | 146 |
| AgU021596_v1.1 | 434 | 23 | b chain principles and pitfalls in designing site directed peptide ligands | AAH30983 | 8.60E-045 | 73 | 120.6 | 100 |
| AgU021787_v1.1 | 431 | 0 | linker for activation of t cells member 2 | AES01005 | 3.41E-069 | 87 | 219.2 | 143 |
| AgU021800_v1.1 | 432 | 4 | immunoglobulin lambda light chain | ABY55575 | 2.11E-063 | 85 | 200.7 | 128 |
| AgU021831_v1.1 | 431 | 9 | ig heavy chain v-iii region vh26-like | XP_510217 | 1.49E-067 | 80 | 213.0 | 141 |
| AgU021926_v1.1 | 429 | 0 | deleted in malignant brain tumors 1 | XP_544052 | 2.46E-073 | 89 | 249.6 | 144 |
| AgU022348_v1.1 | 417 | 0 | wiskott-aldrich syndrome protein | XP_002719911 | 1.90E-059 | 100 | 196.1 | 89 |
| AgU022428_v1.1 | 415 | 0 | phosphatidylinositol 3-kinase regulatory subunit alpha | NP_777000 | 9.72E-070 | 97 | 184.9 | 92 |
| AgU022457_v1.1 | 414 | 0 | neutrophil cytosol factor 4 | XP_002914572 | 5.20E-038 | 83 | 141.4 | 91 |
| AgU022496_v1.1 | 413 | 0 | linker for activation of t-cells family member 1 | XP_002927386 | 3.84E-022 | 97 | 97.1 | 67 |
| AgU022532_v1.1 | 414 | 8 | immunoglobulin kappa light chain v-j region | ADK09631 | 3.19E-063 | 87 | 201.8 | 131 |
| AgU022588_v1.1 | 400 | 14 | immunoglobulin lambda light chain | ABY55575 | 3.21E-049 | 76 | 164.1 | 131 |
| AgU022717_v1.1 | 411 | 1 | beta-2-microglobulin precursor | XP_002913215 | 5.67E-077 | 100 | 234.6 | 113 |
| AgU022894_v1.1 | 404 | 11 | igl@ protein | AAH73769 | 2.66E-029 | 64 | 115.9 | 137 |
| AgU023024_v1.1 | 396 | 0 | immunoglobulin heavy chain variable region vh3 | AAL35302 | 4.05E-037 | 70 | 140.6 | 122 |
| AgU023081_v1.1 | 398 | 0 | cd97 antigen-like | XP_002921043 | 6.44E-048 | 88 | 174.1 | 99 |
| AgU023161_v1.1 | 398 | 0 | mitochondrial antiviral-signaling protein | EFB15451 | 2.91E-045 | 85 | 163.3 | 132 |
| AgU023357_v1.1 | 390 | 0 | immunoglobulin lambda light chain variable region | ABY55580 | 6.00E-056 | 78 | 181.4 | 129 |
| AgU023397_v1.1 | 391 | 4 | immunoglobulin heavy chain variable region | 1NL0_H | 8.34E-044 | 75 | 153.3 | 129 |
| AgU023408_v1.1 | 391 | 16 | immunoglobulin lambda light chain vlj region | BAC01858 | 1.42E-046 | 82 | 127.5 | 82 |
| AgU023412_v1.1 | 390 | 0 | complement component 4 binding beta | AER95313 | 1.83E-061 | 93 | 199.5 | 126 |
| AgU023489_v1.1 | 387 | 19 | immunoglobulin lambda light chain vlj region | AAH12876 | 4.84E-058 | 85 | 190.3 | 116 |
| AgU023540_v1.1 | 386 | 0 | immunoglobulin lambda light chain variable region | XP_855010 | 2.60E-051 | 80 | 171.8 | 121 |
| AgU023562_v1.1 | 386 | 0 | dna cross-link repair 1c (pso2 cerevisiae) | XP_003364407 | 4.90E-085 | 100 | 269.6 | 127 |
| AgU023596_v1.1 | 385 | 0 | toll-like receptor 8 | BAC11412 | 1.16E-075 | 100 | 233.8 | 117 |
| AgU023604_v1.1 | 386 | 0 | atlastin-1 isoform 2 | AER94702 | 9.84E-089 | 100 | 268.5 | 128 |
| AgU023621_v1.1 | 375 | 12 | ig heavy chain v-iii region vh26-like | XP_001149684 | 1.22E-050 | 81 | 168.7 | 122 |
| AgU023747_v1.1 | 381 | 0 | complement c2-like isoform 2 | XP_002929674 | 3.20E-027 | 98 | 114.4 | 52 |
| AgU023798_v1.1 | 383 | 3 | carcinoembryonic antigen-related cell adhesion molecule 1-like | EFB26560 | 1.02E-054 | 86 | 137.5 | 83 |
| AgU023966_v1.1 | 377 | 0 | cytokine receptor common subunit gamma precursor | EFB15757 | 7.16E-079 | 97 | 247.7 | 125 |
| AgU024085_v1.1 | 373 | 0 | 59 kda 2 -5 -oligoadenylate synthase-like protein | EFB23134 | 1.60E-056 | 94 | 193.4 | 124 |
| AgU024129_v1.1 | 372 | 0 | complement component 4 binding beta | AER95313 | 8.06E-048 | 94 | 164.1 | 88 |
| AgU024166_v1.1 | 374 | 2 | c-x-c motif chemokine 2-like | EFB12807 | 1.24E-030 | 96 | 114.8 | 60 |
| AgU024203_v1.1 | 370 | 1 | beta-defensin 1 | AER97104 | 3.93E-033 | 92 | 121.3 | 84 |
| AgU024489_v1.1 | 359 | 0 | t cell receptor beta variable 12 | EFB18057 | 1.92E-049 | 87 | 163.7 | 99 |
| AgU024585_v1.1 | 358 | 0 | interferon-induced protein with tetratricopeptide repeats 3 | XP_003434495 | 5.22E-040 | 89 | 142.5 | 84 |
| AgU024587_v1.1 | 360 | 12 | mhc class i antigen | AAW22618 | 2.47E-059 | 88 | 192.2 | 114 |
| AgU024613_v1.1 | 360 | 0 | transcription factor 12 isoform 1 | AAI51748 | 1.15E-052 | 98 | 181.0 | 119 |
| AgU025020_v1.1 | 340 | 10 | ig lambda chain v region 4a-like protein | AES10776 | 1.66E-026 | 95 | 74.3 | 42 |
| AgU025064_v1.1 | 346 | 13 | immunoglobulin lambda light chain variable region | ABY55563 | 4.71E-045 | 85 | 152.9 | 98 |
| AgU025088_v1.1 | 345 | 2 | immunoglobulin lambda light chain variable region | ABY55557 | 1.95E-050 | 82 | 166.4 | 115 |
| AgU025091_v1.1 | 343 | 0 | cd274 molecule | EFB13833 | 3.25E-073 | 97 | 228.0 | 114 |
| AgU025186_v1.1 | 342 | 0 | c-c motif chemokine 14 | XP_002923672 | 9.20E-036 | 92 | 127.9 | 67 |
| AgU025195_v1.1 | 330 | 0 | immunoglobulin light chain variable region | ADX66093 | 9.89E-025 | 63 | 99.8 | 106 |
| AgU025507_v1.1 | 331 | 0 | b-cell linker protein | EDL41847 | 4.95E-033 | 81 | 128.3 | 86 |
| AgU025533_v1.1 | 333 | 0 | interleukin 1 type ii | XP_002913551 | 4.47E-064 | 95 | 209.9 | 109 |
| AgU025601_v1.1 | 328 | 0 | igl@ protein | EFB23656 | 1.42E-050 | 84 | 166.4 | 106 |
| AgU025691_v1.1 | 325 | 0 | clusterin precursor | EFB22766 | 1.15E-066 | 94 | 216.9 | 110 |
| AgU025722_v1.1 | 322 | 0 | immunoglobulin kappa light chain vlj region | 3QEG_L | 8.99E-030 | 82 | 82.8 | 52 |
| AgU025747_v1.1 | 323 | 0 | yth domain family protein 1 | BAE40338 | 2.61E-073 | 100 | 225.7 | 107 |
| AgU025751_v1.1 | 293 | 1 | immunoglobulin light chain variable region | AAB66566 | 2.46E-042 | 84 | 144.4 | 96 |
| AgU026172_v1.1 | 310 | 0 | immunoglobulin heavy chain variable region | CAE45490 | 2.99E-044 | 78 | 150.2 | 103 |
| AgU026191_v1.1 | 297 | 4 | ig heavy chain v-iii region vh26-like | XP_003778445 | 1.62E-034 | 77 | 129.4 | 84 |
| AgU026218_v1.1 | 306 | 0 | collagen type iv alpha-3-binding protein | XP_002804474 | 3.30E-054 | 98 | 188.3 | 101 |
| AgU026235_v1.1 | 305 | 0 | interleukin-1 family member 10 | XP_854689 | 1.70E-051 | 98 | 169.5 | 82 |
| AgU026375_v1.1 | 286 | 0 | immunoglobulin lambda light chain | ADK09698 | 6.61E-027 | 84 | 107.5 | 66 |
| AgU026441_v1.1 | 293 | 7 | immunoglobulin heavy chain variable region | ABW80088 | 1.74E-024 | 68 | 99.0 | 101 |
| AgU026484_v1.1 | 294 | 6 | immunoglobulin heavy chain variable region | AAQ05550 | 5.34E-043 | 83 | 146.7 | 96 |
| AgU026527_v1.1 | 292 | 2 | immunoglobulin heavy chain variable region | AAA52824 | 1.51E-028 | 73 | 109.4 | 90 |
| AgU026616_v1.1 | 285 | 1 | ig lambda chain v-iii region loi-like | EFB21234 | 1.31E-020 | 72 | 88.6 | 69 |
| AgU026714_v1.1 | 283 | 8 | carcinoembryonic antigen-related cell adhesion molecule 8 | EFB30058 | 2.08E-025 | 83 | 96.7 | 67 |
| AgU026716_v1.1 | 281 | 1 | immunoglobulin heavy chain variable region | AAL68807 | 3.03E-035 | 85 | 125.6 | 83 |
| AgU026727_v1.1 | 280 | 4 | immunoglobulin heavy chain variable region | AAK11751 | 3.43E-038 | 77 | 133.7 | 95 |
| AgU026804_v1.1 | 280 | 0 | immunoglobulin m heavy chain | AAX73309 | 6.55E-057 | 98 | 191.8 | 93 |
| AgU026831_v1.1 | 279 | 0 | immunoglobulin lambda light chain variable region | ABY55575 | 4.97E-037 | 85 | 131.0 | 80 |
| AgU026889_v1.1 | 276 | 11 | anti-tetanus toxoid immunoglobulin heavy chain variable region | ACS96041 | 5.79E-040 | 79 | 138.7 | 92 |
| AgU027006_v1.1 | 273 | 1 | immunoglobulin lambda light chain variable region | EFB23656 | 1.15E-026 | 85 | 104.4 | 67 |
| AgU027049_v1.1 | 270 | 0 | inhibitor of kappa light polypeptide gene enhancer in b- kinase epsilon | AES00135 | 6.74E-040 | 92 | 139.8 | 88 |
| AgU027076_v1.1 | 268 | 18 | immunoglobulin lambda light chain | NP_001077269 | 7.02E-018 | 85 | 62.8 | 40 |
| AgU027229_v1.1 | 262 | 13 | immunoglobulin lambda light chain vlj region | BAC01854 | 9.55E-036 | 83 | 131.7 | 85 |
| AgU027277_v1.1 | 259 | 1 | immunoglobulin gamma heavy chain | AAX73308 | 4.72E-021 | 67 | 92.8 | 83 |
| AgU027282_v1.1 | 260 | 7 | immunoglobulin lambda light chain variable region | EFB23656 | 6.51E-041 | 88 | 140.6 | 84 |
| AgU027294_v1.1 | 258 | 0 | lymphocyte cytosolic protein 2 | AFE69490 | 2.67E-051 | 97 | 173.7 | 85 |
| AgU027332_v1.1 | 257 | 3 | immunoglobulin variable region | ADX65560 | 1.75E-025 | 90 | 101.3 | 72 |
| AgU027339_v1.1 | 252 | 5 | immunoglobulin lambda light chain variable region | AAB94913 | 7.77E-038 | 86 | 132.5 | 76 |
| AgU027427_v1.1 | 252 | 0 | peroxisomal trans-2-enoyl- reductase | XP_003925776 | 1.07E-016 | 86 | 80.5 | 50 |
| AgU027447_v1.1 | 248 | 0 | immunoglobulin lambda light chain variable region | AAB94913 | 1.47E-028 | 90 | 108.6 | 63 |
| AgU027533_v1.1 | 247 | 0 | testosterone-specific single chain antibody fragment | ABY55580 | 2.42E-020 | 79 | 64.3 | 48 |
| AgU027574_v1.1 | 247 | 0 | interleukin-1 receptor-associated kinase 1 | XP_549367 | 7.45E-051 | 98 | 178.3 | 81 |
| AgU027578_v1.1 | 247 | 5 | mhc class i antigen | ABY27242 | 5.34E-044 | 92 | 147.5 | 82 |
| AgU027703_v1.1 | 240 | 0 | cd40 tnf receptor superfamily member 5 | AER95840 | 1.36E-042 | 94 | 149.4 | 79 |
| AgU027709_v1.1 | 222 | 1 | immunoglobulin heavy chain variable region | AAK11761 | 1.55E-034 | 90 | 123.3 | 72 |
| AgU027711_v1.1 | 240 | 0 | immunoglobulin heavy chain variable region | CBH31870 | 5.69E-027 | 95 | 76.3 | 40 |
| AgU027767_v1.1 | 223 | 13 | immunoglobulin heavy chain variable region | AAC18296 | 1.60E-021 | 89 | 90.5 | 48 |
| AgU027776_v1.1 | 237 | 0 | immunoglobulin lambda light chain variable region | EFB23656 | 7.87E-034 | 87 | 122.1 | 73 |
| AgU027799_v1.1 | 215 | 0 | immunoglobulin lambda light chain variable region | CAI99702 | 3.38E-031 | 84 | 114.8 | 71 |
| AgU027845_v1.1 | 235 | 0 | immunoglobulin j chain | EFB23253 | 2.28E-044 | 98 | 149.4 | 75 |
| AgU027896_v1.1 | 229 | 2 | immunoglobulin heavy chain variable region | AEX29442 | 4.07E-022 | 79 | 92.0 | 64 |
| AgU027897_v1.1 | 216 | 0 | immunoglobulin lambda light chain variable region | AAB94913 | 3.99E-020 | 83 | 78.2 | 48 |
| AgU027914_v1.1 | 226 | 0 | immunoglobulin heavy chain variable region | CAL04584 | 2.24E-034 | 85 | 122.9 | 75 |
| AgU027956_v1.1 | 211 | 0 | immunoglobulin heavy chain variable region | BAC01303 | 6.12E-021 | 74 | 88.6 | 70 |
| AgU027994_v1.1 | 213 | 1 | immunoglobulin lambda light chain variable region | EFB23656 | 2.14E-020 | 90 | 87.0 | 50 |
| AgU028082_v1.1 | 224 | 0 | cd274 molecule | EFB13833 | 2.84E-042 | 95 | 147.1 | 74 |
| AgU028104_v1.1 | 220 | 2 | immunoglobulin lambda light chain variable region | EFB23656 | 1.40E-020 | 83 | 87.8 | 55 |
| AgU028108_v1.1 | 223 | 0 | immunoglobulin lambda light chain v-j region | AAB95460 | 3.86E-029 | 84 | 109.8 | 75 |
| AgU028388_v1.1 | 198 | 0 | immunoglobulin lambda light chain | CAJ75506 | 1.87E-017 | 83 | 79.0 | 55 |
| AgU028434_v1.1 | 211 | 2 | mhc class i antigen | AFD64705 | 1.30E-032 | 86 | 117.5 | 69 |
| AgU028666_v1.1 | 197 | 2 | b chain principles and pitfalls in designing site directed peptide ligands | ABY55586 | 2.83E-027 | 89 | 105.1 | 67 |
| AgU028807_v1.1 | 195 | 0 | mhc class ii antigen | AAS45655 | 2.99E-016 | 96 | 78.2 | 63 |
| AgU028829_v1.1 | 195 | 0 | immunoglobulin lambda light chain variable region | ACS83572 | 1.32E-029 | 90 | 114.4 | 62 |
| AgU028845_v1.1 | 194 | 1 | immunoglobulin heavy chain variable region | ADM44055 | 1.73E-020 | 77 | 87.4 | 63 |
| AgU028870_v1.1 | 192 | 0 | hla class ii histocompatibility antigen gamma chain isoform 2 | XP_536468 | 2.05E-037 | 95 | 135.2 | 62 |
| AgU029012_v1.1 | 188 | 0 | signal transducer and activator of transcription 5a | EHH58145 | 4.65E-034 | 100 | 128.6 | 62 |
| AgU029014_v1.1 | 184 | 0 | cd40 tnf receptor superfamily member 5 | AER95840 | 2.87E-020 | 95 | 89.4 | 45 |
| AgU029151_v1.1 | 183 | 1 | immunoglobulin heavy chain variable region | ACI25519 | 2.54E-022 | 86 | 92.0 | 60 |
| AgU029219_v1.1 | 177 | 0 | immunoglobulin lambda chain variable region | ACN59532 | 3.95E-025 | 92 | 98.6 | 53 |
| AgU029281_v1.1 | 176 | 0 | immunoglobulin variable region | AAI42356 | 4.62E-019 | 87 | 85.5 | 54 |
| AgU029367_v1.1 | 160 | 0 | immunoglobulin light chain variable region | EHH62537 | 1.87E-023 | 88 | 94.4 | 52 |
| AgU029368_v1.1 | 176 | 0 | immunoglobulin lambda light chain variable region | CAC10917 | 8.27E-022 | 85 | 89.7 | 57 |
| AgU029412_v1.1 | 174 | 0 | immunoglobulin kappa light chain v-j region | ADK09645 | 2.06E-020 | 58 | 88.2 | 90 |
| AgU029520_v1.1 | 171 | 0 | immunoglobulin heavy chain variable region | CAL04592 | 5.54E-025 | 84 | 97.8 | 57 |
| AgU029597_v1.1 | 169 | 0 | immunoglobulin lambda light chain variable region | ABU90705 | 1.37E-023 | 87 | 94.7 | 56 |
| AgU029671_v1.1 | 166 | 0 | protein artemis | XP_002929927 | 2.63E-030 | 98 | 120.2 | 55 |
| AgU029681_v1.1 | 167 | 2 | immunoglobulin gamma- partial | AAX73307 | 1.81E-030 | 98 | 117.5 | 55 |
| AgU029744_v1.1 | 152 | 6 | immunoglobulin lambda light chain variable region | EFB29065 | 1.29E-017 | 84 | 78.6 | 51 |
| AgU029778_v1.1 | 163 | 0 | granulocyte colony-stimulating factor | EFB23381 | 2.06E-023 | 92 | 96.7 | 54 |
| AgU029787_v1.1 | 164 | 10 | immunoglobulin heavy chain variable region | CAA85560 | 1.45E-023 | 87 | 94.7 | 54 |
| AgU029819_v1.1 | 159 | 1 | anti-tetanus toxoid immunoglobulin heavy chain variable region | AEQ73765 | 1.83E-015 | 91 | 73.2 | 37 |
| AgU029822_v1.1 | 154 | 1 | immunoglobulin heavy chain variable region | ABP98319 | 6.41E-019 | 100 | 82.0 | 41 |
| AgU029864_v1.1 | 160 | 0 | 2 -5 -oligoadenylate synthetase-like | AES03288 | 4.34E-026 | 96 | 107.1 | 53 |
| AgU029877_v1.1 | 160 | 1 | immunoglobulin heavy chain variable region | AAT51717 | 1.39E-021 | 84 | 89.4 | 50 |
| AgU030072_v1.1 | 154 | 0 | mhc class i antigen | ABI96691 | 2.39E-023 | 94 | 95.5 | 51 |
| AgU030136_v1.1 | 152 | 0 | proteasome subunit beta type-10 | AES04860 | 1.35E-024 | 100 | 97.8 | 50 |
| AgU030220_v1.1 | 150 | 0 | anti-tetanus toxoid immunoglobulin light chain variable region | AAG43502 | 1.39E-021 | 91 | 89.0 | 49 |
| AgU030232_v1.1 | 149 | 1 | immunoglobulin heavy chain variable region | XP_003465028 | 3.70E-019 | 87 | 83.6 | 48 |
| AgU030297_v1.1 | 133 | 0 | immunoglobulin heavy chain variable region | AEX29123 | 2.20E-016 | 85 | 75.5 | 41 |
| AgU030334_v1.1 | 146 | 0 | egf-like module mucin- hormone receptor-like 2 | NP_001033756 | 1.78E-023 | 97 | 100.9 | 48 |
| AgU030428_v1.1 | 143 | 2 | immunoglobulin lambda light chain variable region | AAY33420 | 1.93E-015 | 85 | 73.2 | 47 |
| AgU030561_v1.1 | 139 | 0 | collagen type iv alpha-3-binding protein | XP_003759529 | 3.34E-023 | 100 | 100.1 | 46 |
| AgU030648_v1.1 | 133 | 0 | immunoglobulin lambda light chain v-j region | CAC10901 | 1.39E-014 | 84 | 70.1 | 44 |
| AgU030740_v1.1 | 127 | 0 | immunoglobulin lambda chain | EFB23656 | 1.38E-015 | 87 | 73.6 | 41 |
| AgU030915_v1.1 | 128 | 0 | lymphocyte cytosolic protein 2 | 2EAP_A | 3.24E-020 | 97 | 84.7 | 42 |
| AgU032084_v1.1 | 2781 | 0 | poliovirus receptor-related 3 | XP_002917364 | 0 | 99 | 1000.7 | 501 |
| AgU032087_v1.1 | 2523 | 1 | 2 -oligoadenylate synthetase 2 | XP_002925285 | 0 | 90 | 731.5 | 406 |
| AgU032107_v1.1 | 1529 | 1 | bactericidal permeability-increasing protein | XP_002915210 | 0 | 91 | 795.0 | 483 |
| AgU032109_v1.1 | 1498 | 0 | glucose-6-phosphate isomerase | XP_002925234 | 0 | 99 | 989.2 | 479 |
| AgU032113_v1.1 | 1442 | 0 | dipeptidyl peptidase 4 | XP_002924912 | 0 | 98 | 945.7 | 458 |
| AgU032133_v1.1 | 1248 | 0 | serpin b13 | XP_002917546 | 1.21E-121 | 96 | 369.8 | 186 |
| AgU032164_v1.1 | 1272 | 10 | immunoglobulin alpha heavy chain | BAC87503 | 0 | 77 | 545.4 | 433 |
| AgU032193_v1.1 | 832 | 17 | mhc class ii antigen | AAM89237 | 9.43E-163 | 97 | 463.8 | 253 |
| AgU032201_v1.1 | 804 | 1 | complement c3-like | XP_002927812 | 6.47E-161 | 97 | 498.4 | 252 |
| AgU032210_v1.1 | 771 | 12 | immunoglobulin kappa light chain vlj region | AAC37305 | 4.95E-093 | 86 | 283.1 | 178 |
| AgU032243_v1.1 | 645 | 0 | proteasome subunit beta type-10 | XP_002918479 | 1.48E-125 | 98 | 367.1 | 185 |
| AgU032247_v1.1 | 1123 | 49 | immunoglobulin gamma- partial | AAA51281 | 7.68E-131 | 81 | 394.4 | 288 |
| AgU032259_v1.1 | 610 | 1 | immunoglobulin gamma- partial | AAA30965 | 2.72E-022 | 88 | 100.9 | 62 |
| AgU032269_v1.1 | 582 | 17 | immunoglobulin heavy chain | AAS85994 | 1.52E-070 | 80 | 222.6 | 168 |
| AgU032276_v1.1 | 574 | 0 | complement component 6 | EFB14077 | 1.07E-068 | 95 | 234.6 | 123 |
| AgU032285_v1.1 | 543 | 19 | ig heavy chain v-iii region vh26-like | XP_003778445 | 1.91E-045 | 86 | 122.1 | 72 |
| AgU032290_v1.1 | 538 | 16 | immunoglobulin heavy chain variable region subgroup 3 | AAA69734 | 6.39E-068 | 79 | 214.9 | 151 |
| AgU032291_v1.1 | 738 | 12 | immunoglobulin lambda light chain | S25738 | 5.93E-119 | 84 | 350.1 | 228 |
| AgU032302_v1.1 | 528 | 10 | immunoglobulin heavy chain variable region subgroup 3 | BAJ83709 | 7.33E-062 | 81 | 157.5 | 103 |
| AgU032311_v1.1 | 496 | 11 | immunoglobulin heavy chain | AAA69729 | 1.33E-051 | 82 | 125.6 | 81 |
| AgU032318_v1.1 | 485 | 9 | immunoglobulin heavy chain variable region | AAM89701 | 2.65E-031 | 60 | 120.2 | 162 |
| AgU032319_v1.1 | 486 | 26 | ig heavy chain v-iii region vh26-like | AAA69729 | 5.02E-074 | 81 | 229.6 | 158 |
| AgU032325_v1.1 | 529 | 18 | immunoglobulin heavy chain | XP_003814082 | 1.11E-041 | 90 | 107.5 | 62 |
| AgU032327_v1.1 | 471 | 10 | igl@ protein | AAH73769 | 1.20E-054 | 81 | 169.9 | 119 |
| AgU032333_v1.1 | 893 | 23 | mhc class i antigen | AAC83173 | 1.07E-156 | 85 | 452.2 | 294 |
| AgU032336_v1.1 | 449 | 7 | immunoglobulin lambda light chain variable region | ABY55563 | 1.70E-030 | 75 | 116.7 | 93 |
| AgU032347_v1.1 | 444 | 19 | immunoglobulin lambda light chain variable region | AAO46065 | 6.40E-068 | 82 | 213.8 | 145 |
| AgU032349_v1.1 | 432 | 6 | immunoglobulin lambda light chain | S25756 | 3.54E-054 | 75 | 180.6 | 134 |
| AgU032350_v1.1 | 859 | 8 | immunoglobulin gamma 1 heavy chain constant region | AAL35302 | 9.41E-076 | 83 | 186.8 | 126 |
| AgU032353_v1.1 | 425 | 14 | ig heavy chain v-iii region vh26-like | XP_510217 | 3.94E-046 | 88 | 121.7 | 71 |
| AgU032361_v1.1 | 688 | 8 | ig heavy chain v-iii region vh26-like | XP_510217 | 7.04E-075 | 84 | 235.3 | 148 |
| AgU032364_v1.1 | 400 | 7 | immunoglobulin lambda light chain vlj region | AAH73769 | 2.23E-039 | 93 | 107.5 | 59 |
| AgU032380_v1.1 | 388 | 25 | immunoglobulin v lambda chain | EFB23656 | 1.05E-056 | 86 | 183.0 | 115 |
| AgU032382_v1.1 | 967 | 6 | igh-6 protein | EFB26488 | 1.02E-135 | 93 | 403.7 | 227 |
| AgU032389_v1.1 | 385 | 22 | mhc class i antigen | ABY27206 | 6.97E-065 | 86 | 211.5 | 122 |
| AgU032395_v1.1 | 372 | 0 | complement factor h-like | XP_002931261 | 2.33E-057 | 81 | 188.3 | 122 |
| AgU032411_v1.1 | 336 | 10 | immunoglobulin lambda light chain vlj region | BAC01855 | 4.40E-045 | 89 | 157.1 | 113 |
| AgU032419_v1.1 | 396 | 4 | immunoglobulin lambda light chain variable region | ABY55575 | 8.40E-052 | 84 | 170.6 | 124 |
| AgU032421_v1.1 | 318 | 0 | immunoglobulin lambda light chain variable region | CAG27376 | 4.82E-034 | 92 | 115.2 | 66 |
| AgU032445_v1.1 | 238 | 3 | immunoglobulin heavy chain variable region | AAR38567 | 3.35E-026 | 88 | 101.7 | 59 |
| AgU032461_v1.1 | 332 | 0 | immunoglobulin lambda light chain variable region | ABA70822 | 1.16E-030 | 71 | 115.5 | 111 |
| AgU032464_v1.1 | 282 | 12 | immunoglobulin kappa chain variable region | AAF09245 | 6.66E-042 | 91 | 147.1 | 83 |
| AgU032476_v1.1 | 271 | 4 | immunoglobulin kappa chain variable region | CAJ75549 | 9.84E-034 | 84 | 122.1 | 77 |
| AgU032482_v1.1 | 317 | 8 | immunoglobulin lambda light chain | AER46340 | 8.45E-044 | 86 | 149.1 | 105 |
| AgU032484_v1.1 | 404 | 8 | immunoglobulin lambda light chain variable region | AAO46065 | 5.74E-047 | 82 | 123.6 | 87 |
| AgU032487_v1.1 | 483 | 15 | immunoglobulin heavy chain variable region subgroup 3 | AAM89748 | 5.33E-046 | 91 | 121.3 | 72 |
| AgU032489_v1.1 | 302 | 11 | immunoglobulin heavy chain variable region subgroup 3 | BAJ83717 | 5.52E-028 | 85 | 82.4 | 47 |
| AgU032498_v1.1 | 593 | 24 | immunoglobulin lambda light chain vlj region | AAH73769 | 4.40E-064 | 82 | 208.4 | 160 |
| AgU032518_v1.1 | 251 | 7 | immunoglobulin heavy chain variable region | BAI52119 | 2.54E-027 | 81 | 105.5 | 66 |
| AgU032525_v1.1 | 924 | 34 | mhc class i antigen | AAC83173 | 1.53E-156 | 90 | 452.2 | 294 |
| AgU032528_v1.1 | 243 | 7 | immunoglobulin heavy chain variable region | EFB23107 | 1.11E-021 | 90 | 90.5 | 52 |
| AgU032544_v1.1 | 478 | 18 | immunoglobulin lambda light chain variable region | XP_854941 | 4.95E-040 | 94 | 96.3 | 53 |
| AgU032564_v1.1 | 218 | 3 | immunoglobulin heavy chain variable region | ACS95883 | 2.51E-024 | 82 | 97.4 | 67 |
| AgU032572_v1.1 | 215 | 1 | interferon-induced transmembrane protein 3-like | DAA24046 | 1.61E-042 | 97 | 144.8 | 71 |
| AgU032583_v1.1 | 410 | 10 | immunoglobulin lambda light chain variable region | AAH22098 | 6.74E-048 | 71 | 164.5 | 140 |
| AgU032593_v1.1 | 202 | 6 | immunoglobulin lambda light chain | AAY16636 | 1.95E-021 | 90 | 90.1 | 62 |
| AgU032607_v1.1 | 411 | 13 | immunoglobulin lambda light chain variable region | ABY55580 | 2.03E-042 | 84 | 108.2 | 69 |
| AgU032643_v1.1 | 439 | 8 | igl@ protein | EFB23656 | 1.89E-064 | 91 | 203.4 | 115 |
| AgU032667_v1.1 | 361 | 6 | immunoglobulin lambda light chain variable region | ABY55575 | 1.21E-050 | 88 | 167.2 | 106 |
| AgU032720_v1.1 | 188 | 13 | immunoglobulin heavy chain variable region | AAM87988 | 1.40E-022 | 80 | 91.3 | 62 |
| AgU032757_v1.1 | 171 | 6 | immunoglobulin heavy chain variable region | CAL04915 | 1.50E-017 | 97 | 78.6 | 40 |
| AgU032765_v1.1 | 440 | 10 | immunoglobulin lambda light chain | ABY55575 | 3.11E-062 | 86 | 198.0 | 121 |
| AgU032788_v1.1 | 382 | 17 | immunoglobulin lambda-2b light chain variable region | EFB23656 | 2.05E-061 | 91 | 194.5 | 115 |
