# Supplementary material for: Transcriptome of the dead: characterisation of immune genes and marker development from necropsy samples in a free-ranging marine mammal
Source: BMC Genomics. 2013 Jan 24;14:52. doi: 10.1186/1471-2164-14-52 (PMC3563519; doi:10.1186/1471-2164-14-52)
Supplement: Additional file 2: Table S2 — The ten most commonly expressed sequences (in order of abundance) with associated BLAST matches. Data are presented sequentially for each type of tissue. Contig names correspond to the tissue-specific assemblies. Immune-related transcripts are denoted by an 'X'. [file 1471-2164-14-52-S2.docx]

**Table S2:** The ten most commonly expressed sequences (in order of abundance) with associated BLAST matches. Data are presented sequentially for each type of tissue as well as for the assembly based on the combined 454 data. Immune-related transcripts are denoted by an 'X'.

| Tissue type | Contig ID | Length (bp) | No of reads | Description | Immune-related? | Species | Common name | E-value |
| --- | --- | --- | --- | --- | --- | --- | --- | --- |
| Testis | contig00007 | 2949 | 8124 | testin |  | *Mus musculus* | mouse | 8e^-13^ |
|  | contig00018 | 2506 | 5758 | hypothetical protein |  | *Bos Taurus* | bull | 9e^-96^ |
|  | contig01543 | 470 | 5140 | ATP synthase subunit beta |  | *Medicago truncatula* | clover | 3e^-40^ |
|  | contig00671 | 845 | 4425 | hypothetical protein |  | *Melampsora larici populina* | poplar leaf rust | 4e^-42^ |
|  | contig02097 | 101 | 1783 | tubulin beta-2C chain |  | *Heterocephalus glaber* | naked mole rat | 0.0 |
|  | contig00263 | 1215 | 284 | tubulin beta-4B chain |  | *Mus musculus* | mouse | 0.0 |
|  | contig01920 | 200 | 284 | polyubiquitin |  | *gallus gallus* | chicken | 2e^-38^ |
|  | contig00082 | 1720 | 278 | eukaryotic translation elongation factor 1 alpha 1 variant |  | *Homo sapiens* | human | 0.0 |
|  | contig00141 | 1474 | 233 | tubulin alpha-3 chain |  | *Gallus gallus* | chicken | 0.0 |
|  | contig01877 | 228 | 213 | polyubiquitin-C isoform 1 |  | *Sus scrofa* | wild boar | 1e^-34^ |
| Heart | contig00023 | 1496 | 5442 | hypothetical protein |  | *Medicago* | clover | 9e^-54^ |
|  | contig00024 | 1493 | 5424 | hypothetical protein |  | *cavia porcellus* | guinea pig | 1e^-72^ |
|  | contig00008 | 2062 | 5035 | hypothetical protein |  | *Bos taurus* | bull | 4e^-79^ |
|  | contig00003 | 2841 | 2484 | NADH dehydrogenase subunit 1 |  | *Zalophus californius* | California sea lion | 1e^-107^ |
|  | contig00019 | 1543 | 1694 | Testin |  | *Mus musculus* | mouse | 1e^-13^ |
|  | contig00002 | 3634 | 935 | NADH dehydrogenase subunit 5 |  | *Arctocephalus pusillus* | Cape fur seal | 0.0 |
|  | contig00151 | 469 | 887 | cytochrome c oxidase subunit I |  | *Arctocephalus pusillus* | Cape fur seal | 6e^-75^ |
|  | contig00014 | 1656 | 671 | cytochrome c oxidase subunit III |  | *Arctocephalus pusillus* | Cape fur seal | e^-113^ |
|  | contig00015 | 1655 | 639 | NADH dehydrogenase subunit 4 |  | *Arctocephalus forsteri* | Cape fur seal | e^-120^ |
|  | contig00176 | 187 | 442 | cytochrome c oxidase subunit I |  | *Arctocephalus pusillus* | Cape fur seal | 3e^-22^ |
| Spleen | contig00880 | 803 | 3491 | hypothetical protein LOC100559304 |  | *Anolis carolinensis* | Carolina anole | 3e^-28^ |
|  | contig02824 | 221 | 3244 | immunoglobulin gamma heavy chain B | X | *Canis lupus familiaris* | dog | 1e^-31^ |
|  | contig00179 | 1553 | 3102 | Ig mu chain C region | X | *Canis lupus familiaris* | dog | 1e^-106^ |
|  | contig02696 | 259 | 2050 | hypothetical protein MTR_5g051110 |  | *Medicago truncatula* | clover | 2e^-32^ |
|  | contig03110 | 138 | 1362 | hypothetical protein BOS_23207 |  | *Bos taurus* | cow | 5e^-18^ |
|  | contig00383 | 1151 | 1308 | Ig alpha-2 chain C region | X | *Homo sapiens* | human | 1e^-137^ |
|  | contig02557 | 321 | 1298 | hypothetical protein BOS_23226 |  | *Bos taurus* | cow | 2e^-56^ |
|  | contig00064 | 2107 | 1227 | Heat shock 70 kDa protein 1B |  | *Bos taurus* | cow | 0.0 |
|  | contig00175 | 1573 | 1167 | immunoglobulin J chain isoform 1 | X | *Canis lupus familiaris* | dog | 6e^-80^ |
|  | contig00033 | 2585 | 1123 | NADH dehydrogenase subunit 1 |  | *Zalophus californianus* | California sea lion | 1e^-105^ |
| Intestine | contig00916 | 120 | 18761 | LRRG00134 |  | *Rattus norvegicus* | rat | 2e^-12^ |
|  | contig00604 | 422 | 9685 | unnamed protein product |  | *Mus musculus* | mouse | 8e^-37^ |
|  | contig00741 | 259 | 9111 | Tar1p |  | *Medicago truncatula* | clover | 1e^-17^ |
|  | contig00491 | 497 | 3285 | hypothetical protein LOC100559304 |  | *Anolis carolinensis* | lizard | 8e^-29^ |
|  | contig0010 | 1017 | 2934 | Ig alpha-2 chain C region | X | *Homo sapiens* | human | 1e^-126^ |
|  | contig00120 | 950 | 2029 | NADH dehydrogenase subunit 1 |  | *Zalophus californianus* | California sea lion | 4e^-54^ |
|  | contig00840 | 178 | 1769 | immunoglobulin lambda-like polypeptide 5-like | X | *Canis lupus familiaris* | dog | 1e^-23^ |
|  | contig00410 | 549 | 978 | uncharacterized protein LOC310926 |  | *Rattus norvegicus* | rat | 1e^-73^ |
|  | contig00159 | 842 | 845 | ATP synthase F0 subunit 6 |  | *Pusa caspica* | Caspian seal | 2e^-70^ |
|  | contig00834 | 183 | 840 | unnamed protein product |  | *Mus musculus* | mouse | 5e^-25^ |
| Kidney | contig04067 | 357 | 8669 | CHK1 checkpoint-like protein |  | *Helicoverpa armigera* | cotton bollworm | 1e^-15^ |
|  | contig04947 | 104 | 8397 | hypothetical protein BOS_23230 |  | *Bos taurus* | cow | 1e^-12^ |
|  | contig04693 | 156 | 6664 | hypothetical protein BOS_1871 |  | *Bos taurus* | cow | 4e^-26^ |
|  | contig00951 | 998 | 5871 | NADH dehydrogenase subunit 1 |  | *Arctocephalus pusillus* | Cape fur seal | 1e^-54^ |
|  | contig04552 | 192 | 5764 | hypothetical protein |  | *Oryctolagus cuniculus* | European rabbit | 5e^-12^ |
|  | contig01876 | 729 | 4887 | hypothetical protein |  | *Oryctolagus cuniculus* | European rabbit | 2e^-16^ |
|  | contig04490 | 212 | 3715 | hypothetical protein LOC100559304 |  | *Anolis carolinensis* | lizard | 2e^-21^ |
|  | contig02747 | 567 | 3287 | cytochrome c oxidase subunit I |  | *Petrogal exanthopus celeris* | wallaby | 2e^-56^ |
|  | contig04118 | 334 | 3208 | rRNA external transcribed spacer |  | *Mus musculus* | mouse | 6e^-14^ |
|  | contig04665 | 162 | 3114 | ATP synthase F0 subunit 6 |  | *Zalophus californianus* | California sea lion | 2e^-14^ |
| Lung | contig01090 | 464 | 10566 | hypothetical protein MTR_5g051110 |  | *Medicago truncatula* | clover | 2e^-35^ |
|  | contig01291 | 232 | 7437 | hypothetical protein BOS_23207 |  | *Bos taurus* | cow | 2e^-22^ |
|  | contig01171 | 405 | 6972 | hypothetical protein BOS_23226 |  | *Bos taurus* | cow | 6e^-61^ |
|  | contig01436 | 113 | 6182 | hypothetical protein LOC100559304 |  | *Anolis carolinensis* | lizard | 3e^-11^ |
|  | contig01143 | 430 | 2925 | rRNA promoter binding protein |  | *Rattus norvegicus* | rat | 8e^-75^ |
|  | contig01165 | 413 | 1643 | hypothetical protein LOC100559304 |  | *Anolis carolinensis* | lizard | 4e^-29^ |
|  | contig00048 | 2106 | 1016 | Heat shock 70 kDa protein 1B |  | *Bos taurus* | cow | 0.0 |
|  | contig00082 | 1741 | 589 | eukaryotic translation elongation factor 1 |  | *Pan troglodytes* | chimpanzee | 0.0 |
|  | contig01406 | 133 | 527 | hypothetical protein LOC100717127 |  | *Cavia porcellus* | guinea pig | 1e^-17^ |
|  | contig00475 | 821 | 515 | unknown |  | *Zea mays* | corn | 1e^-13^ |
| Skin | contig19264 | 197 | 8454 | cytochrome c oxidase subunit III |  | *Martes pennanti* | fisher | 2e^-21^ |
|  | contig19888 | 155 | 7056 | cytochrome c oxidase subunit III |  | *Arctocephalus townsendi* | Guadelupe fur seal | 9e^-18^ |
|  | contig12493 | 479 | 7048 | cytochrome c oxidase subunit II |  | Zalophus *californianus* | California sea lion | 4e^-73^ |
|  | contig19948 | 152 | 6054 | zymogen granule protein 16 homolog B |  | *Canis lupus familiaris* | dog | 2e^-14^ |
|  | contig19959 | 151 | 4928 | cytochrome c oxidase subunit I |  | *Vicugna pacos* | alpaca | 8e^-19^ |
|  | contig19158 | 205 | 4909 | hypothetical protein |  | *Neisseria polysaccharea* | giant panda | 4e^-23^ |
|  | contig20348 | 129 | 3744 | cytochrome c oxidase subunit I |  | *Hippotragus niger* | sable antelope | 5e^-16^ |
|  | contig01306 | 1644 | 3667 | NADH dehydrogenase subunit 4 |  | *Arctocephalus forsteri* | Australian fur seal | 1e^-127^ |
|  | contig19212 | 199 | 3653 | cytochrome c oxidase subunit I |  | *Arctocephalus pusillus* | Cape fur seal | 1e^-14^ |
